# Teaching the process of science with primary literature: Using the CREATE pedagogy in ecological courses

**DOI:** 10.1002/ece3.9644

**Published:** 2022-12-21

**Authors:** Kevin G. Smith, Christopher J. Paradise

**Affiliations:** ^1^ Departments of Biology and Environmental Studies Davidson College Davidson North Carolina USA

**Keywords:** CREATE pedagogy, evidence‐based teaching, inclusive teaching, science practice skills, undergraduate biology education

## Abstract

There have been numerous calls for improved pedagogical practices in biological education, and there is a clear need for such improvements in ecology and related curricula. Most ecology‐related texts lack pedagogy and are designed to be content‐rich. National initiatives, such as *Vision & Change,* provide guidance on undergraduate biology education, including increasing use of evidence‐based active learning, and taking a more conceptual and science practice skills approach. Biology education research is rich with evidence‐based teaching practices, which reveal that active learning approaches implemented in thoughtful ways lead to strong learning gains relative to lecture‐based course delivery. CREATE (Consider, Read, Elucidate the hypothesis, Analyze and interpret data, Think of the next Experiment) integrates evidence‐based active pedagogical practices into one approach to STEM education that focuses heavily on the process of science and science practice skills rather than content delivery by replacing the textbook with selected journal articles. The approach focuses on deep reading and analysis of primary literature; immersing students in the literature is an advantage of the pedagogy. CREATE was developed and tested in other biological disciplines (genetics and molecular biology) that have long been at the forefront of pedagogical best practices in biology. We transformed two upper‐level undergraduate ecological courses (Conservation Biology, and Biodiversity and Ecology) into CREATE courses. We provide examples of assignments, student work, and assessments of the approach, illustrating the various ways CREATE can be successfully implemented. The approach can be adopted in part, to ease into it and test it out, or in whole. We recommend that ecology teachers consider making their courses more active, if they have not already done so; adopting pedagogical practices embedded within CREATE can be a way to achieve active learning. The CREATE approach and other evidence‐based pedagogical best practices lead to strong learning gains and more inclusive learning environments.

## Abstract 2

National initiatives, such as Vision & Change, provide guidance on undergraduate biology education, including increasing use of evidence‐based active learning. CREATE (Consider, Read, Elucidate the hypothesis, Analyze and interpret data, Think of the next Experiment) integrates evidence‐based active pedagogical practices into one approach that focuses on the process of science, science practice skills, and deep reading of the primary literature. We transformed two upper‐level undergraduate ecological courses (Conservation Biology, and Biodiversity and Ecology) into CREATE courses and provided examples of assignments, student work, and assessments, illustrating the various ways in which CREATE can be successfully implemented. The CREATE approach and other evidence‐based pedagogical best practices lead to strong learning gains and more inclusive learning environments.

## 1 INTRODUCTION—BRINGING VISION & CHANGE TO THE ECOLOGY CLASSROOM

A critical challenge in higher education is facilitating the adoption of evidence‐based pedagogical practices (D'Avanzo et al., 2006; Kenyon et al., 2019). There is substantial evidence that active, inquiry‐based, and inclusive teaching approaches are more effective for student learning as compared to a traditional lecture that prioritizes content delivery (Armbruster et al., 2009; D'Avanzo, 2003; Freeman et al., 2014; Haak et al., 2011). As a result of this evidence, over the past decade, there have been several reports encouraging faculty to shift their teaching toward pedagogical practices that facilitate the participation of a greater percentage of students, that is they are more inclusive, and also better equip students for the modern biology that is practiced today. Modern biology is a discipline that benefits from a deep understanding of core concepts, highly developed competencies, and a recognition that most biology problems are interdisciplinary and draw on other STEM fields, the social sciences, and the humanities (American Association for the Advancement of Science, 2011; Harvey et al., 2016; National Research Council, 2009).

Ecology faculty, like those of many other STEM disciplines, have struggled with the challenge of updating our pedagogical practices (D'Avanzo, 2003). As students, most of today's tenured and mid‐career faculty themselves took courses that were overwhelmingly comprised of lectures with an emphasis on content coverage with little or no time for exploration, knowledge construction, and discussion of topics outside of, but relevant to, biology, including social implications and inclusive practices such as decolonizing ecology (de Vos, 2020; Trisos et al., 2021). Further, an embarrassing reality of graduate training is that few STEM faculty have had any formal pedagogical training by the time they become professors (Love Stowell et al., 2015; Tanner & Allen, 2005). Finally, textbooks reflect and perpetuate these issues. Most biology textbooks overwhelmingly emphasize content coverage and the presentation of “facts,” therefore focusing student attention on the *products* of science and not the *process* of science (Barsoum et al., 2013; Duncan et al., 2011; Hoskins & Stevens, 2009; Smith, 2018), with the latter approach advocated by *Vision and Change*, a national call to transform undergraduate biology education (American Association for the Advancement of Science, 2011). Thus, texts rarely take advantage of or facilitate best practices in teaching and learning, leading to a disconnect between pedagogical research and teaching materials. In summary, faculty interested in changing their pedagogical practices are working against several forms of inertia: their experience as students, lack of formal pedagogical training, limits of the teaching materials at their disposal, and lack of institutional support for such change (e.g., release time to redesign courses; Henderson & Dancy, 2007).

Many ecology teachers and texts include social implications in their ecology‐related courses, but in our experience, those implications are often limited to issues such as the impacts of global climate change or the conversion of land by human activities on biodiversity or ecological systems. Often missing from those discussions is the disproportionate impact of environmental issues on underrepresented peoples, perspectives of diverse peoples inhabiting the varied ecosystems around the world, and better representation of underrepresented groups in the field of ecology. Marginalized communities bear the brunt of environmental impacts due to racial and economic disparities, regional differences in pollution (Yip, 2021), and even the impact of systemic racism on ecology and conservation of urban flora and fauna (Schell et al., 2020). Yet Indigenous Peoples, for instance, have relationships with the environment and cultural perspectives that offer other, equally valid ways of knowing (Kimmerer, 2012; Louis, 2007; Trisos et al., 2021) that can help students understand ecological and conservation concepts. Further, those underrepresented in STEM, such as Black Ecologists, are not well‐represented in textbooks but make important contributions to ecology and conservation science, such as expanding the concept of keystone species to include culturally important plant species (Coe & Gaoue, 2020) or making unrecognized contributions to tropical ecology (de Vos, 2020). Highlighting such scientists by selecting their papers allows students from those groups to see themselves as ecologists and leads to increased inclusivity in ecologically relevant courses (Schinske et al., 2016).

How can today's ecology faculty, including visiting professors, instructors, and professors across all ranks, overcome these substantial challenges? We suspect that most faculty are at least minimally aware of the above challenges; they are discussed by peers, professional organizations, federal agencies, future employers, educational experts, and internally at institutions of higher education themselves. Advice and strategies for incorporating and adopting evidence‐based teaching approaches appear occasionally in the ecological literature (e.g., D'Avanzo, 2003; Ebert‐May et al., 2006; Nordlund, 2016) but that may not translate into a change in the classroom. In our view, faculty are frequently told what their teaching should do, but this is rarely translated into how faculty can achieve these goals in the day‐to‐day business of teaching in the classroom or laboratory.

## 2 ONE METHOD TO ACHIEVE MODERN PEDAGOGICAL GOALS: CREATE

One approach to addressing the pedagogical deficiencies found in many textbooks in ecology courses is to dispense with conventional teaching materials—textbooks—altogether. In our case, we have done this by applying the CREATE (Consider, Read, Elucidate the hypothesis, Analyze and interpret data, Think of the next Experiment) teaching method (Hoskins et al., 2007) to two courses focused on ecological concepts: “Conservation Biology and Biodiversity” (CBB) and “Ecology,” each taught at Davidson College in North Carolina, USA, a small liberal arts college. The CREATE method is based almost entirely on the primary literature, with the goal of focusing on the process of science but offering opportunities to highlight the work of BIPOC Ecologists. In our cases, our courses no longer focus on teaching students *about* ecology and conservation biology, but rather focus on students learning *how to do* ecology and conservation biology, as represented by the primary professional literature. Professors who “do” ecology find their field exciting, interesting, and relevant. Structuring their courses around other ecologist's research allows them to convey their own excitement about the research and discoveries. We have found this approach to be energizing to both us and our students.

Although the CREATE method was originally designed and implemented within the context of genetics and cell biology (Hoskins et al., 2007), the method is easily modified and applied to other fields in which the scientific process and peer‐reviewed literature are central to the profession. However, we are aware of only one published case study of the application of CREATE to courses outside of cell and molecular biology, chemistry, and engineering (Beck, 2019). Beck (2019) describes a modified jigsaw approach using CREATE‐style assignments in an ecology lecture‐only course. Further, the recently published 4DEE framework for ecology education has “ecology practice” as one dimension of the framework but offers no guidance on how to address this important dimension in the classroom (Berkowitz et al., 2018). This suggests that many instructors in our fields of interest—ecology, conservation, evolution, and behavior—may not be aware of tools that can help address modern pedagogical goals as expressed in 4DEE. CREATE is one such approach. Even for those who choose not to adopt the full CREATE approach, our experience suggests that many instructors will find something within the method that can benefit them and the students in their courses.

## 3 SUMMARY OF THE CREATE APPROACH

Detailed expositions on the CREATE method have been published elsewhere (Hoskins et al., 2007; Hoskins & Krufka, 2015; Hoskins & Stevens, 2009), but we provide an overview here focused on our applications. CREATE focuses on replicating, in the classroom, how the scientific process plays out for most professional biologists: through the lens of discipline‐based primary literature. Journal articles are presented to students in a piecemeal fashion and are initially stripped of identifying (author, title, journal) or summarizing (abstract) information. We provide students with general advice on reading scientific literature, which includes how to take notes effectively and read different sections of scientific papers for distinct purposes (see Appendix S1). The goal is to facilitate students' own processing and analysis of raw information in the paper, unbiased by the authors' interpretations and conclusions. As a result, students have an opportunity to experience how scientific knowledge is generated and develop their own science practice skills (Hoskins & Krufka, 2015; Stevens & Hoskins, 2014). Beyond developing science practice skills, however, this practice‐centered learning approach helps students see themselves as competent scientists capable of generating knowledge themselves (Rosemond et al., 2020) and contributing to more inclusive and equitable classroom STEM education.

Often, the first step in a CREATE module is for students to read a paper's introduction with the title and abstract redacted or removed. We use PDF editors to redact content (see Appendix S1). In our experience, redaction is an important part of the process, as summaries such as the title and abstract often contain the authors' primary conclusions. The goal is *deep reading and analysis* of the introduction, with an emphasis on understanding how past studies, established theories, or practical problems lead to the development of hypotheses or questions and to define key terms and connections between them in preparation for concept mapping (Hoskins et al., 2007). Introductions often convey complex information in equally complex or convoluted sentences and paragraphs, which may need significant decoding and deconstruction. We provide students with the time and tools to conduct this analysis outside of class, and then recap and review in class.

Important tools in this process include concept mapping and annotation of the introduction via note‐taking and highlighting on the documents themselves, either as paper or electronic copies. We find that concept mapping (Novak, 1990) is a particularly useful tool for understanding key concepts. Ecological principles, theories, and laws (such as they exist), are notorious for their contingency, nonlinearity, and complexity (Johnson & Lidström, 2018). Ecological concepts are therefore challenging to understand in simple, linear, or hierarchical frameworks. For example, a bullet‐point list summarizing the productivity–diversity relationship cannot convey its full complexity and contingency (e.g., Borer et al., 2014; van Ruijven & Berendse, 2005). By contrast, concept mapping embraces complexity and systems thinking. This approach allows students to synthesize multiple factors, actors, and ideas and directly address how they affect each other via feedbacks and direct and indirect effects. Concept maps may be based on an organizing question (“Why are there more species in the tropics?”) or a prediction or thesis statement (“Habitat heterogeneity increases diversity.”).

Reading an Introduction facilitates metacognitive reflection and often leads to rich concept maps. We sometimes assign introductions that are only three paragraphs long. Others may be five pages long. In our experience, the depth of learning does not correlate with the length of the reading. In fact, we have found that decoding the very short introductions found in some papers is an advanced skill that provides students with practice in metacognition. Students are asked to consider questions such as, “What do I know and how do I know it?” “Do I understand this idea?,” “What do I not understand about this idea?” and “What else do I need to know to understand this idea?” Answering questions such as these facilitate the integration of knowledge and understanding of the process of their own learning. Practice with metacognition is an important end goal itself and its mastery is an essential step toward self‐regulated and self‐directed learning (Mariano et al., 2021; Tanner, 2012). Additionally, concept mapping facilitates the construction of knowledge across the course, as concepts will be encountered multiple times over the course of the semester, even when they are not the focus of the research at hand (e.g., “competition” may be embedded in an introduction to papers about succession, habitat selection, or resource utilization).

By the end of the Introduction, students should have constructed an understanding of the supporting theory and goals of the study. As a result, students are prepared to deconstruct the methods—how do the authors aim to achieve these goals? We ask students to cartoon or diagram the workflow of the methods or specific aspects of the methods such as the experimental design or data collection procedures. An important task for students is mapping specific methods to the associated research questions, goals, or hypotheses addressed by those methods. This can be assigned before class or it can be done in small groups during class, followed by a whole class discussion.

Cartooning ensures that students are prepared to understand the paper, but more broadly, it helps them uncover the fundamentals of experimental design, which is an acknowledged weakness in undergraduate biology education (Dasgupta et al., 2014). A follow‐up step asks students to predict or anticipate results from these methods, which can also be done in small groups during class. For example, students can be asked to draw predicted results under competing hypotheses, either on existing, prelabeled axes (a blank plot) or when given the more advanced task of developing the entire figure themselves, including defining dependent and independent variables and choosing an appropriate visualization. Again, this task links several key aspects of the scientific process: articulation of a hypothesis or question and how the question can be answered with data.

The Results section is often where we spend the most class time. Focusing on results in figures and tables, rather than text, can be a powerful way for students to learn the scientific process (Round & Campbell, 2013), especially when guided thoughtfully by the professor. With the CREATE method, we focus students on identifying the source of the data, decoding or relabeling the axes, identifying the hypothesis or question that the figure addresses, correspondence between the methods and the resulting data, and what we conclude from the figure. These figure annotations may lead to a discussion of outliers, thresholds, or unacknowledged patterns. This also is where a deeper discussion of statistics can often take place, including key concepts such as *p*‐values, sample sizes, variance, and distributions. In some sessions, a side‐lecture on statistical analyses, such as ordination or regression, is necessary to enable students to fully interpret figures.

When students make their own anticipated results figures, a natural follow‐up is to compare their predictions with the actual results in the paper. A key here is that students should not view their figures as “wrong.” Some aspects of student figures may be based on alternative conceptions, but those often will be revised by the students themselves or their peers. Students' guesses at reality may not be supported, but this is simply a refutation of a hypothesis. Interestingly, we have found that many students develop figures that are very different from those in the papers. Sometimes these are superior in terms of data visualization, other times they address interesting alternative hypotheses ignored by the authors.

Before providing students with the Discussion section, we often discuss the goal of synthesizing the results within the context of the Introduction: “If you were to write the Discussion, what would your main conclusions be? What data support your conclusions? How do the results inform the paper's primary question or hypothesis?” This could come in the form of a list of key points. Again, this may be followed with a direct comparison of the students' conclusions and authors' Discussion sections. We have found that early in the semester students attempt to predict what the authors have written. This seems to be based on a misconception of the authors as an absolute authority, a perspective that undermines student agency and self‐efficacy as developing scientists themselves. We have found that careful word choice, (e.g., “What do *you* conclude?” vs. “What do you think the *authors* will conclude?”) is critical to ensuring students approach these assignments as participants in the scientific process and not as passive outside observers of science, as done by others.

Although the Discussion formally concludes the piecemeal reading of each paper in a CREATE course, other follow‐up assignments for synthesis may be appropriate depending on the paper, course, students, and timing within the semester. Some examples of assignments that focus on synthesis include revision of the original concept map but now including the results; a new concept map of the results and how they relate to each other and to the authors' conclusions; a concept map of how the paper fits in with other papers read during the semester.

To illustrate the recursive and continuing nature of scientific exploration, the final step in the analysis of a CREATE paper is for students to design a future experiment (Hoskins et al., 2007; Hoskins & Krufka, 2015). This task, which for us is often an in‐class small group activity, allows students to integrate some of their acquired knowledge, including background information, experimental design, and specific methods, with the more creative side of science. The use of small groups also facilitates inclusivity, as many students who hesitate to participate in in‐class discussions feel more comfortable participating in small groups. We have also integrated this step of the process within mock grant panels, which provides opportunities for peer review and education on the collaborative culture of science and the specifics of how funding bodies operate.

The above cycle, which may last as few as two or three class sessions or as many as five or six, is repeated throughout the semester. Related papers may be grouped in “modules” on related topics, which is a modification of the original CREATE design, in which a sequence of papers from a single laboratory is read and deconstructed (Hoskins et al., 2007).

## 4 CASE STUDIES

Owing to the lack of case studies of teaching approaches in ecology, here we will provide some details and examples from two courses in which we use the CREATE method, Conservation Biology & Biodiversity (CBB, taught by KGS) and Ecology (taught by CP). Each course is open to students who have completed introductory biology or environmental science courses, generally leading to a mixture of second, third, and fourth‐year biology and environmental studies students. For each course, the class size is capped at 32 students. A brief description of each course is provided, followed by how each instructor incorporates the CREATE pedagogy into their courses. More detailed information on the structure and selection of papers for each course is provided in the Appendix S1.

### 4.1 Conservation biology & biodiversity

This course focuses on biodiversity and conservation and is divided into five primary modules: what is conservation biology; the creation, maintenance, and measurement of biodiversity; the value and functions of biodiversity; biodiversity change and loss; and biodiversity management and restoration. Each module is composed of 3–8 journal articles serving as the primary content. Content coverage is not the goal of the course, which instead focuses on a deep understanding of the key principles, ideas, and approaches used in the study of biodiversity and its conservation. As a result, students in this course miss some conservation content likely to be deemed essential by someone somewhere, for example, maximum sustainable yield, effective population size calculations, or a detailed history of the North American model of wildlife conservation. What students gain, instead, are (a) transferable and generalizable skills in scientific practice (e.g., core competencies of V&C; AAAS, 2011) and (b) and self‐constructed knowledge of key themes and principles that appear across the diverse biodiversity and conservation literature, as identified by KGS (Table 1a).

**TABLE 1:** Themes of conservation biology and biodiversity (a) and foundational concepts of ecology (b).

**a**. Example themes emerging from the content taught in a CREATE version of Conservation Biology & Biodiversity
Diversity is a scale‐dependent concept that applies across levels of biological organization. Most species (or other biological types) are rare and few are common. Diversity of resources begets diversity of species/biological types. Sampling effects play important roles in the measurement of biodiversity, in biodiversity loss, and in conservation. Diversity may be beneficial because of mechanisms associated with variation per se (e.g., dilution effect, complementarity), or because of the presence of particular species (e.g., keystone species, sampling effects). The definition of success in conservation relies on values, which are not universally held and agreed upon. Humans are not separate from nature, nature is not pristine, and human influence and well‐being must be part of conservation.
**b**. Foundational concepts emerging from the content taught in a CREATE version of Ecology.
The structure of ecological systems, from individuals to ecosystems, defines the function of ecological systems. Ecological systems grow and are regulated by intrinsic and extrinsic factors. The mechanisms of evolution, including mutation, selection, genetic drift, and gene flow, have led to the diversity of life. Ecological systems, from individuals to ecosystems, are interconnected and interacting. Ecological systems are dynamic and change over space and time. Systems can change but may also maintain a dynamic steady state. Energy flow and nutrient cycling are fundamental properties of ecological systems. Biological processes and dynamic interactions among system components are scale‐dependent.

### 4.2 Ecology

This course focuses on ecological concepts and is also divided into five primary modules: global change ecology; ecosystems; individuals; populations; and communities. Each module includes 2–4 journal articles serving as the primary content, with approximately 1 week spent on each paper. As with CBB, content coverage is not the goal, and significant class time is spent on concept mapping, cartooning methods, figure annotations, and identifying the key points of the discussion. Ecology focuses on foundational concepts and approaches to the study of the distribution and abundance of organisms. Again, as with CBB, students are not exposed to some content considered essential by some teachers, for example, models of predator–prey dynamics, the Allee effect, or detailed descriptions of every nutrient cycle. The foci on the process of science and thinking like an ecologist lead to student gains in the: (a) transferable and generalizable skills in scientific practice (e.g., core competencies of [AAAS, 2011]) and (b) self‐constructed knowledge of foundational concepts that appear across the diverse ecological literature, a set of foundational concepts identified by the instructor, CP (Table 1b).

### 4.3 Example work: general annotations of papers

**FIGURE 1:**
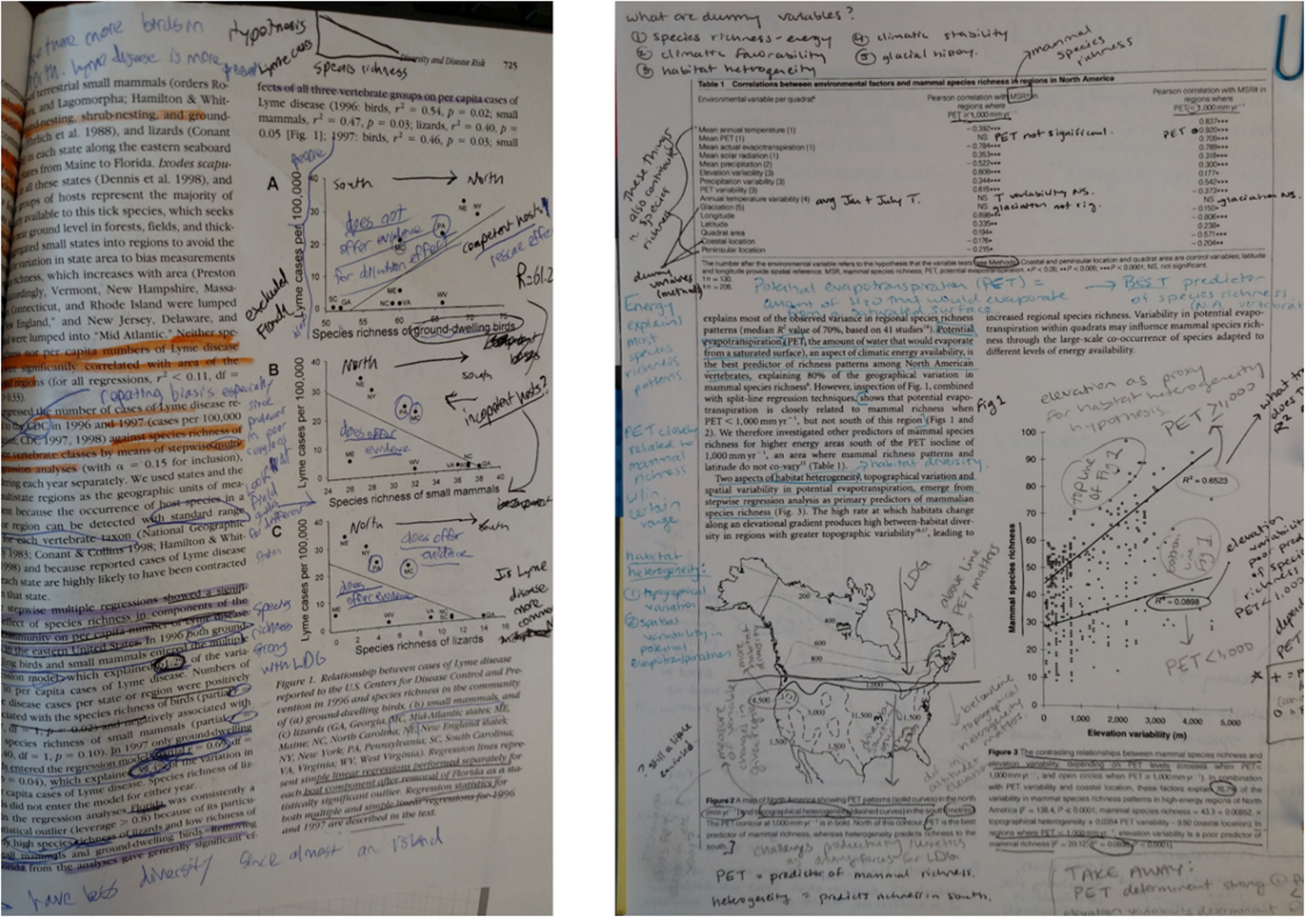
Example student annotations from two papers associated with a module on biodiversity patterns and the value and function of biodiversity in CBB (left: [Ostfeld & Keesing, 2000]; right: [Kerr & Packer, 1997]). Student engagement in the readings is seen in highlighting and underlining, comments in the margins, and annotation of figures and tables. Notably, many students return to previous readings to add new annotations, seen as notes taken with different writing implements

The emphasis on deep reading and paper annotation helps students develop the overlooked skill of reading scientific papers. This is accomplished by breaking papers into small sections, providing students with specific prompts, questions, or goals for their reading, and providing the time for deep reading and annotation. Through these tasks, students gain practice in drawing meaning from the scientific literature. The absence of lectures provides students with the more flexible and creative time, which allows them to make connections with other content, identify areas of weak understanding through metacognition, and engage in active learning, even when working on their own. Student products, sometimes checked for credit or used as assessments of learning, are evidence of the high level of reading engagement that the CREATE approach inspires (Figure 1).

### 4.4 Example work: concept mapping

The experience of reading an introduction leads to potentially complex concept maps that integrate terms, phenomena, and concepts from other parts of the course and that are not directly part of the topic at hand. This often leads to unpredictable and rich discussions as students integrate their ecological knowledge, including related equity and environmental justice issues. As the course proceeds, students build upon their knowledge as they see concepts and terms used in different situations in an integrative fashion. There is an emphasis on deep reading and annotation of papers by section.

**FIGURE 2:**
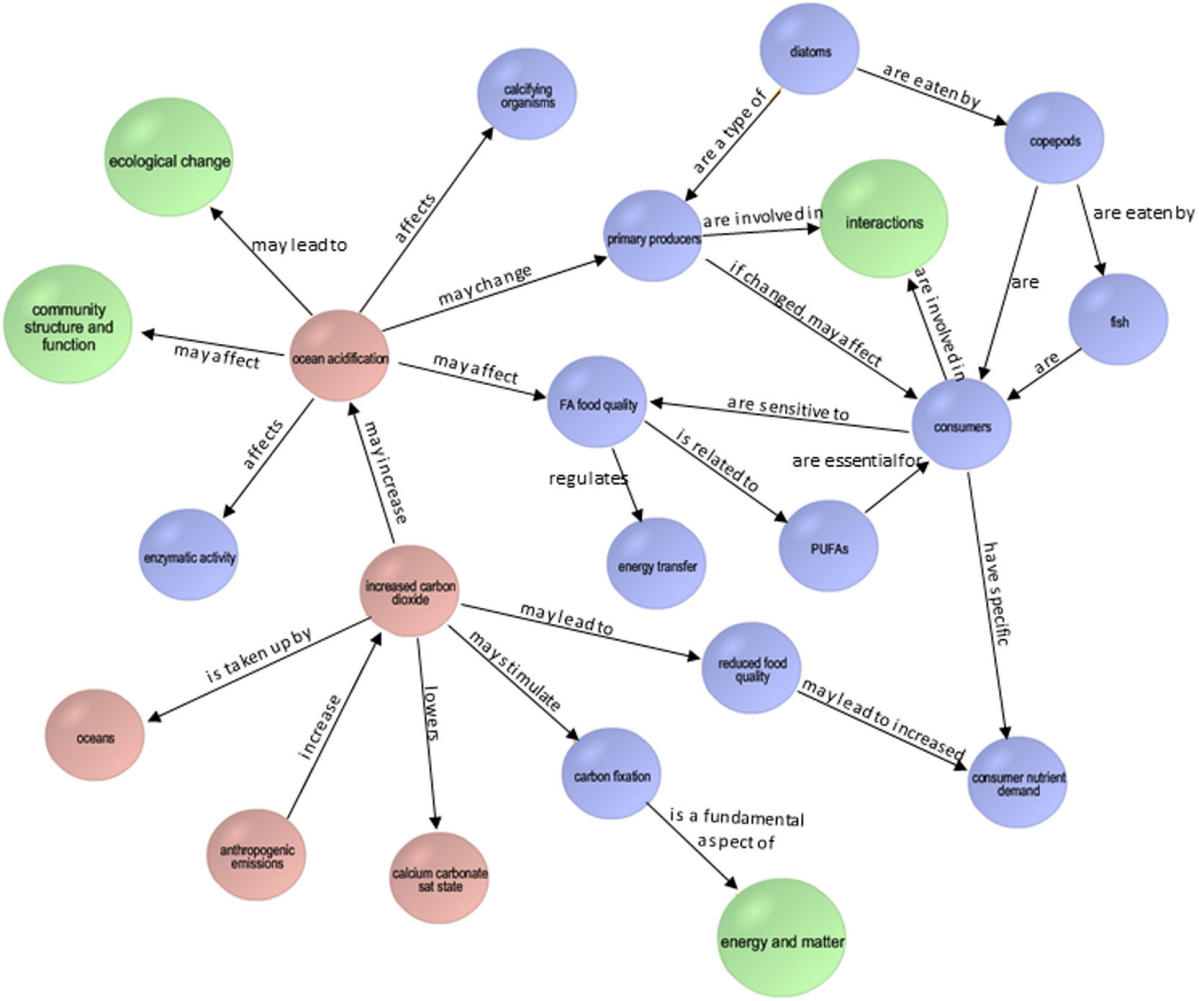
Example of a concept map constructed during an ecology class, examining Rossoll et al. (2012). Foundational concepts are larger green circles, and abiotic and biotic factors are pink and blue, respectively, to help students organize their concepts. In this class‐constructed concept map, student groups had constructed maps, after which a member from each group called out nodes and links, which CP used to construct this map. The free simplemapper.org is no longer supported

In Ecology, CP presents topics as questions. For example, “How is global climate change affecting ocean food webs and energy flow?” In one version of a concept map created for this question, using Rossoll et al. (2012) that studies the effects of ocean acidification on fatty acid composition and resulting trophic transfer, one can see the integration of four foundational concepts of ecology (Table 1b) that CP uses in this course. In Figure 2 (which used the now‐defunct simplemapper.org to construct concept maps in class or prior to class), foundational concepts are large green circles, and abiotic and biotic factors are color‐coded (pink and blue here, respectively), to help students organize their concepts. Other concept mapping tools exist, such as the Concept Connector (Luckie et al., 2011).

### 4.5 Example work: cartooning methods

**FIGURE 3:**
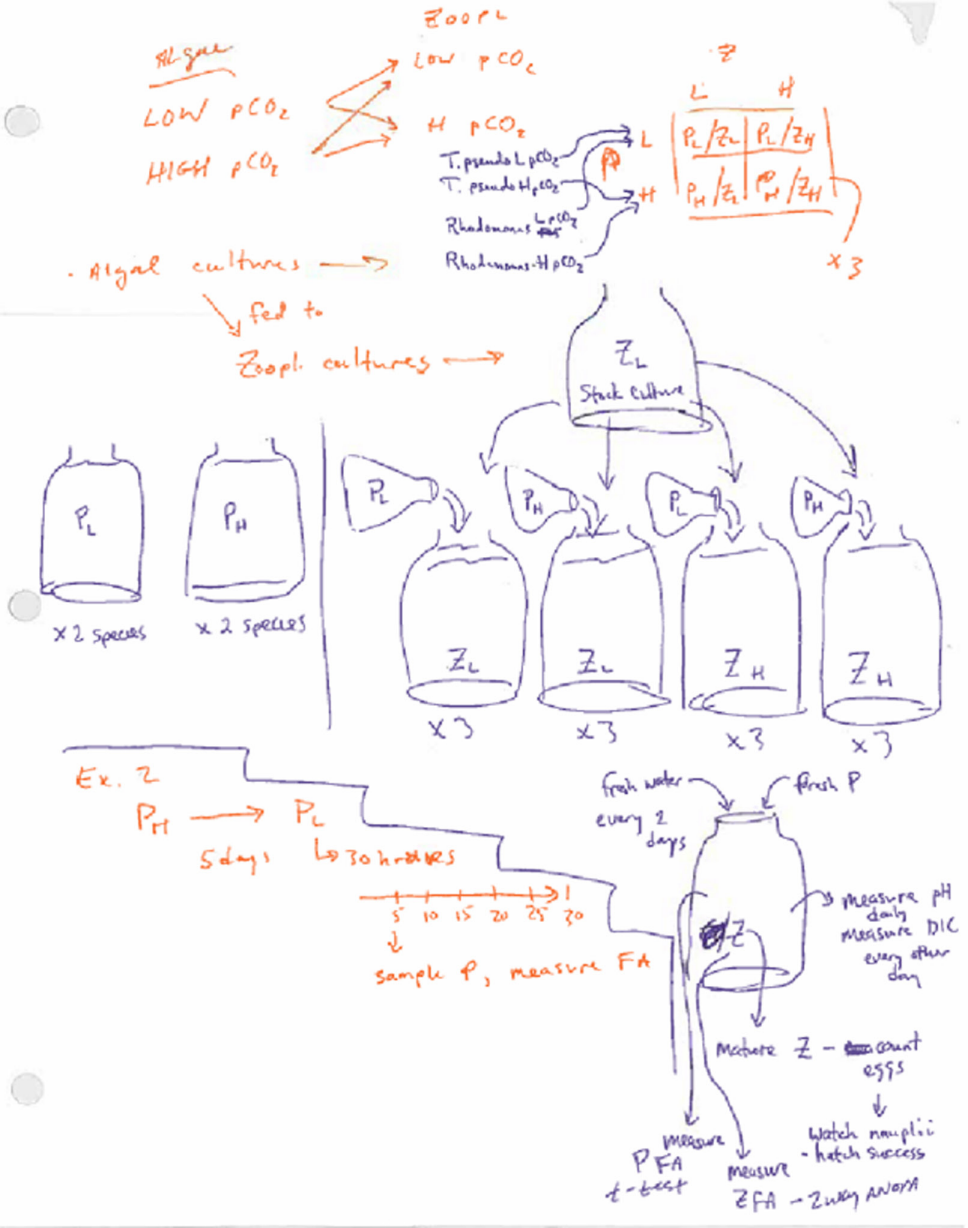
Example of cartooning methods in ecology from Rossoll et al., 2012, an experiment examining the effects of increased pCO_2_ on ocean acidification and the resulting changes in the fatty acid composition of algae and the zooplankton that consume the algae

Cartooning of methods can be done individually by students prior to class, in small groups during class, or by the entire class directing the teacher what to draw on the whiteboard; CP uses all of these approaches, depending upon the topic of the paper and the difficulty of the methods. KGS assigns cartooning outside of class, typically. Figure 3 shows an example of how an ecology class interpreted and drew the complex description of methods in Rossoll et al. (2012); in this case, the figure was drawn by CP as students described what to draw. In general, the level of engagement with experimental design, with some explanation by the instructor of complex statistical methods, facilitates a better understanding of how results should be interpreted. In our experience, students are better able to interpret figures and produce more in‐depth figure annotations when they can connect the methods to the results.

### 4.6 Example work: figure annotations

**FIGURE 4:**
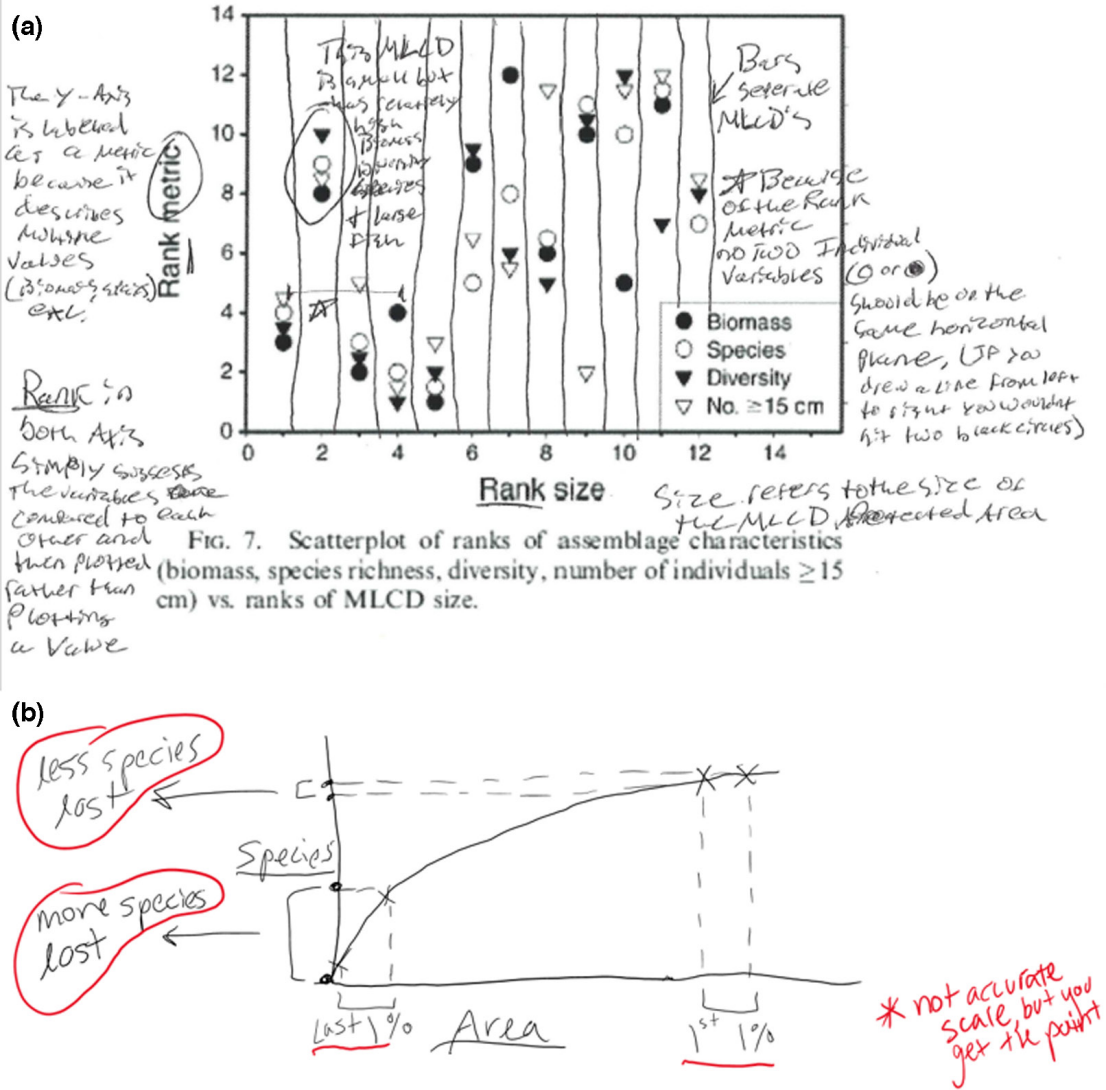
Student annotations of figures in CBB. (a) Annotations of a figure from Friedlander et al. (2007). This student focused on decoding axis labels. (b) One student's response to a prompt to use the species–area relationship to predict whether more species are lost in the first or last phases of habitat destruction

In CBB, KGS often assigns figure annotation as out‐of‐class preparatory work. This task challenges students to create knowledge from quantitative information, identify patterns and thresholds, consider correlation and causation, and learn about analytical and statistical methods used in biology, rather than learning about these skills separate from the practice of science itself. As a result, in‐class discussions of the same results are deep and focused on student questions, hypotheticals, and statistical topics. Students also develop self‐efficacy, moving from “I have no idea what's going on with this figure” to discovering that they can decode figures and glean large amounts of information from them. For example, one student emphasized decoding obtuse axis labels (Figure 4a; ranked size of marine reserves on the x‐axis and ranked fish diversity metrics on the y‐axis). While some students focus on figure presentation, other students will focus on drawing conclusions from the figure (e.g., larger reserves tend to have greater diversity metrics, Figure 4a). This diversity in what students focus on outside of the class translates into breadth and depth of learning in the class during recap discussions with peers.

**FIGURE 5:**
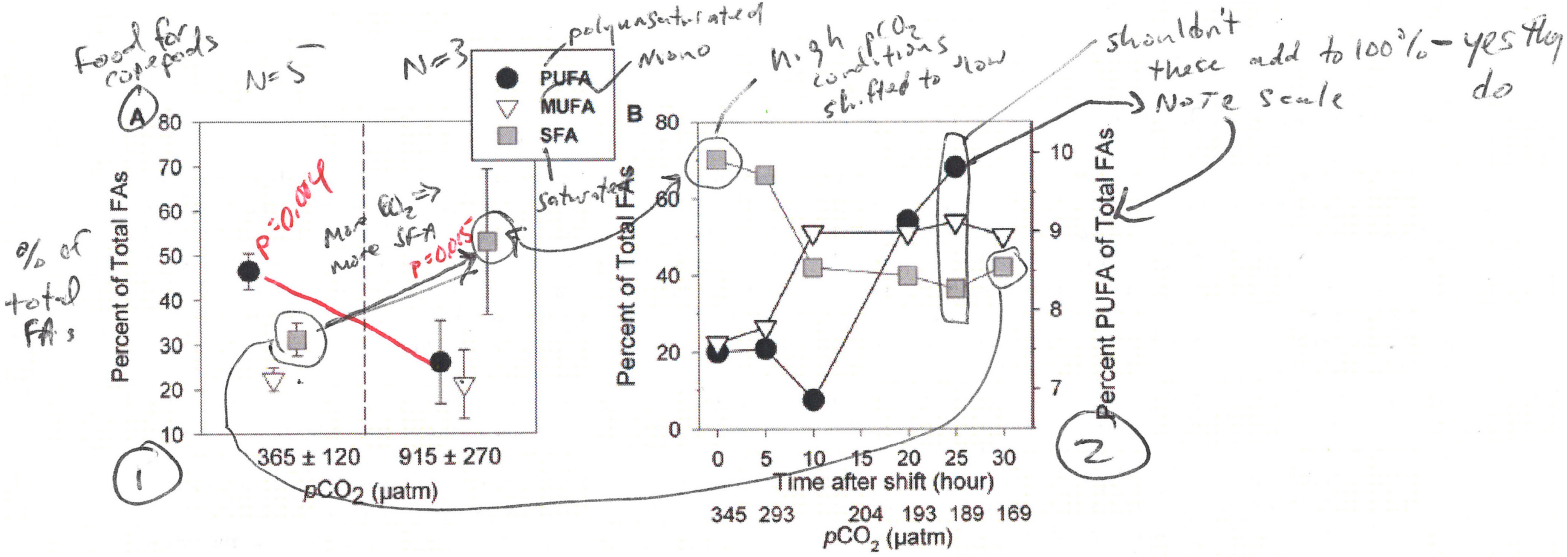
Example of figure annotation in ecology from Rossoll et al., 2012, Figure 1, where annotations decode the figures, highlight statistical and biological results and sample sizes, and show relationships between the two figures

A figure from Rossoll et al. (2012) (Figure 5) shows an example of how the figure was annotated as part of an Ecology class discussion. Students worked in groups to annotate a series of figures from the paper, and then in discussion, directed the teacher (CP) to annotate. This allows discussion of why particular aspects of the figures were annotated, what was or may have been confusing, and what key results were gleaned from the analysis. By performing this task for the entire class, all students have an opportunity to participate and come to a common understanding.

Across a semester, students have opportunities to annotate dozens of figures, compare their annotations with peers, display their work to the whole class for discussion, and work through model figure annotations led by the instructor. This extensive practice with figure annotation prepares students for an advanced science practice skill: drawing their ideas, hypotheses, and predictions as figures. For novices, this task is particularly challenging but is an area in which students can quickly gain skills with guidance and practice. For this reason, KGS often uses “draw a figure that…” assignments as assessments of students' theoretical understanding, quantitative reasoning abilities, and ability to distill complex ideas into formal, assessable predictions, i.e., hypotheses. In one example, KGS asked students to use the species–area relationship to predict whether more species would be lost from the early or late phases of habitat destruction (Figure 4b). As graduate students, some of us may recall being told, “If you can't draw it out, you don't have a hypothesis,” yet how many of us were formally trained in the skills needed to draw out complex and testable hypotheses?

### 4.7 Example work: developing key points/designing the next experiment

Key Points, along with integration across papers and units, may be included in the discussion at the end of a case study or after results annotation (prior to students reading the discussion). Integration is critical for students to connect concepts across papers and units, and often occurs productively in small group discussion. The key points in Table 2a are taken from two such class discussions, which CP wrote on the board as students dictated them. They are shown in the order they were brought up by students and are mildly edited for clarity here. The order and phrasing of key points vary from class to class, and this is an important point about CREATE—the listing of key points and the discussion generated are *student‐driven* and focused on student outcomes, not coverage of content. In addition to key points, CP varies assignments for the discussion of a paper by using prompts to facilitate the integration and consideration of the next steps (Table 2b), as well as social and environmental justice issues. A discussion of the effects of ocean acidification on marine food webs leads to a discussion of the disproportionate impact of human activities on livelihoods, food security, and marginalized coastal communities and nations. Wealthy nations such as the United States disproportionately contribute to greenhouse gas emissions, while coastal communities in poor nations disproportionately suffer the impacts.

**TABLE 2:** Example of key points generated in‐class discussion (a), along with integration across units (b), which facilitates the construction of knowledge.

**a**. Key points generated in the discussion of Rossoll et al. (2012) with Ecology students in two different years.
Key points—Fall 2016 The shift in production of fatty acids occurs rapidly. This causes Z_H_/P_L_ treatment to be similar to the Z_H_/P_H_ treatment and Z_L_/P_H_ to be similar to Z_L_/P_L_. Ecologically, Z_H_/P_H_ and Z_L_/P_L_ are more important than crossed treatments. High CO_2_ leads to lower pH. High CO_2_ changes fatty acid composition and decreases egg production and delays the development of copepods. Some essential fatty acids are in lower concentrations in high CO_2_ treatments. Ocean acidification affects consumer growth and production by affecting the nutritional quality of primary producers. Effects ramify throughout the food web. Key points—Fall 2018 Elevated CO_2_ represents ecological change and leads to reduced pH. Elevated CO_2_ leads to decreases in polyunsaturated fatty acids and increases in saturated fatty acids. Essential fatty acids are affected. Changes in the fatty acid composition of phytoplankton alter zooplankton fatty acid composition and total amount of fatty acids. The change in the fatty acids occurs rapidly, then stabilizes. Dynamics are important in populations with short life cycles and rapid turnover. Food quality for zooplankton is potentially lower at high pCO_2_ and low pH. This could affect the entire marine food web. Phytoplankton and zooplankton at normal (low) pCO_2_ had high polyunsaturated fatty acids and may be an adaptation to current conditions. Egg production and development of zooplankton affected by high pCO_2_ conditions.
**b**. Integration of knowledge and next steps: students may be asked to discuss one or more of the following prompts. Make a connection between either of the first two articles in the global climate change unit and this one. Connect this research paper with any of the foundational concepts that CP uses as a conceptual framework in his Ecology course (evolution, growth and regulation, interactions, energy and matter, ecological change, structure and function, and scale). Predict what might happen to fish‐eating copepods in waters affected by ocean acidification. What is the next experiment, given our discussion of key points? What are the big‐picture conclusions regarding the ecological effects of global climate change?

*Note*: In a, both years are shown to illustrate variation experienced each time the course is taught.

Finally, for a paper at the end of a unit, CP often asks students to make connections to earlier papers, foundational concepts, or the next experiments that researchers could perform to answer questions that arose in the paper. Designing the next experiment is typically done in small groups during class, after which groups describe their experimental designs to the class and the class critiques each design.

### 4.8 Assessments: conservation biology and biodiversity

As part of his participation in National Science Foundation Division of Undergraduate Education grants NSF DUE 1021443 and DUE 1524779, KGS assessed the first offering of his course using pre‐post student self‐assessment of their learning gains, or SALG, approach (Carroll, 2010; Seymour et al., 2000). The assessment results reported here were based on assessments that were approved by the Davidson College HSIRB (protocols 2013‐139 and 2018‐004). The SALG survey focuses on identifying changes in students' perceptions about their own understanding, attitudes, skills, and metacognitive practices using Likert‐style questions associated with specific statements (Table 3). Students were asked about the degree to which each statement applies to their learning, behavior, and self‐beliefs. As a result, SALG results can provide insight into students' self‐perceived learning and self‐perception and sense of self‐efficacy with science skills. Additional discussion of the specific questions in the SALG survey appears in Kenyon et al. (2016).

**TABLE 3:** Example statements used to measure self‐assessed student understanding, attitudes, metacognition, and skills associated with CBB

Category	Example statements *Presently, I…*
Understanding	*…understand how to annotate figures*. *…understand how to work effectively in small groups* *…understand how to look at data and figure out what question the study that generated the data was addressing*
Skills	*…can critically read and analyze journal articles* *…design a study or experiment that follows up on one I read about* *…identify patterns in data*
Attitudes	*…am confident that I can decode data presented in graphs, tables, or charts*. *…confident that I can design a good experiment or study* *…enthusiastic about careers in biology research*
Metacognition	*…am in the habit of connecting key ideas that I learn in my classes with other knowledge* *…am in the habit of thinking about whether I am fully understanding what I am reading* *…am in the habit of thinking about how I know what I know while studying*

*Note*: Summary results are shown in Figure 6.

**FIGURE 6:**
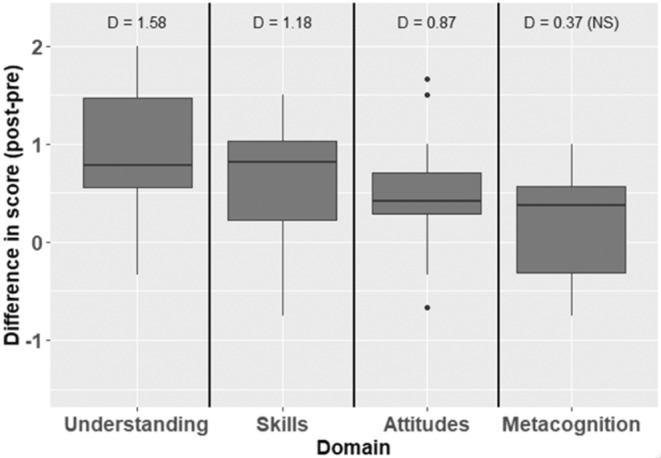
Student assessment of their learning gains (SALG) results from a 2014 implementation of the CREATE method in a Conservation Biology and Biodiversity course at Davidson college. Statistical results are based on within‐subject *t*‐tests, *n* = 20 students who completed the pre‐ and postcourse assessments. Values above each pre‐post comparison are Cohen's *D* effect sizes for the difference in scores within individual students based on the difference between the pre‐ and postcourse assessments

Based on SALG results, students in CBB experienced significant gains in understanding of the scientific process (within‐subject *t*‐test, *t* = 6.4907, *df* = 19, *p*‐value <.0001), skills associated with the scientific process (*t* = 3.8793, *df* = 19, *p*‐value = .001), and attitudes about their ability and interest in science (*t* = 5.2638, *df* = 19, *p*‐value <.0001), and but not metacognition (*t* = 1.6524, *df* = 19, *p*‐value = .1149) (Figure 6). Across the first three domains, students' postcourse scores indicated that, on average, they felt agreement or strong agreement with the statements in Table 4. Their postcourse self‐assessment of metacognition was more neutral and variable with respect to the statements in that domain, however.

**TABLE 4:** Mean (SD) student assessment of their learning gains (SALG) across four domains for pre‐course and post‐course assessments in the 2014 offering of CBB.

Category	Pre‐course score	Post‐course score
Understanding	3.32 (0.61)	4.16 (0.41)
Skills	3.63 (0.69)	4.27 (0.45)
Attitudes	3.77 (0.73)	4.25 (0.44)
Metacognition	3.64 (0.65)	3.86 (0.59)

*Note*: Responses were scored on a 5‐point Likert scale where 0 = complete disagreement with the statement (“not at all”) and 5 = complete agreement with the statement (“a great deal”).

Author KGS also assessed student learning in the ability to analyze and synthesize a key learning outcome of the course, a synthetic understanding of the concept of “biodiversity.” To assess this, KGS provided students with a prompt to generate a concept map of “biodiversity” using as many concepts and terms and labeled connections as they wished. Students were given blank paper and 30 min to complete the same concept map assessment at the beginning and end of CBB. After the course, KGS assessed students' anonymized concept maps for three key metrics: number of relevant concepts, number of correctly labeled connections among concepts, and total number of connections (sum of correctly labeled connections and unlabeled connections).

**FIGURE 7:**
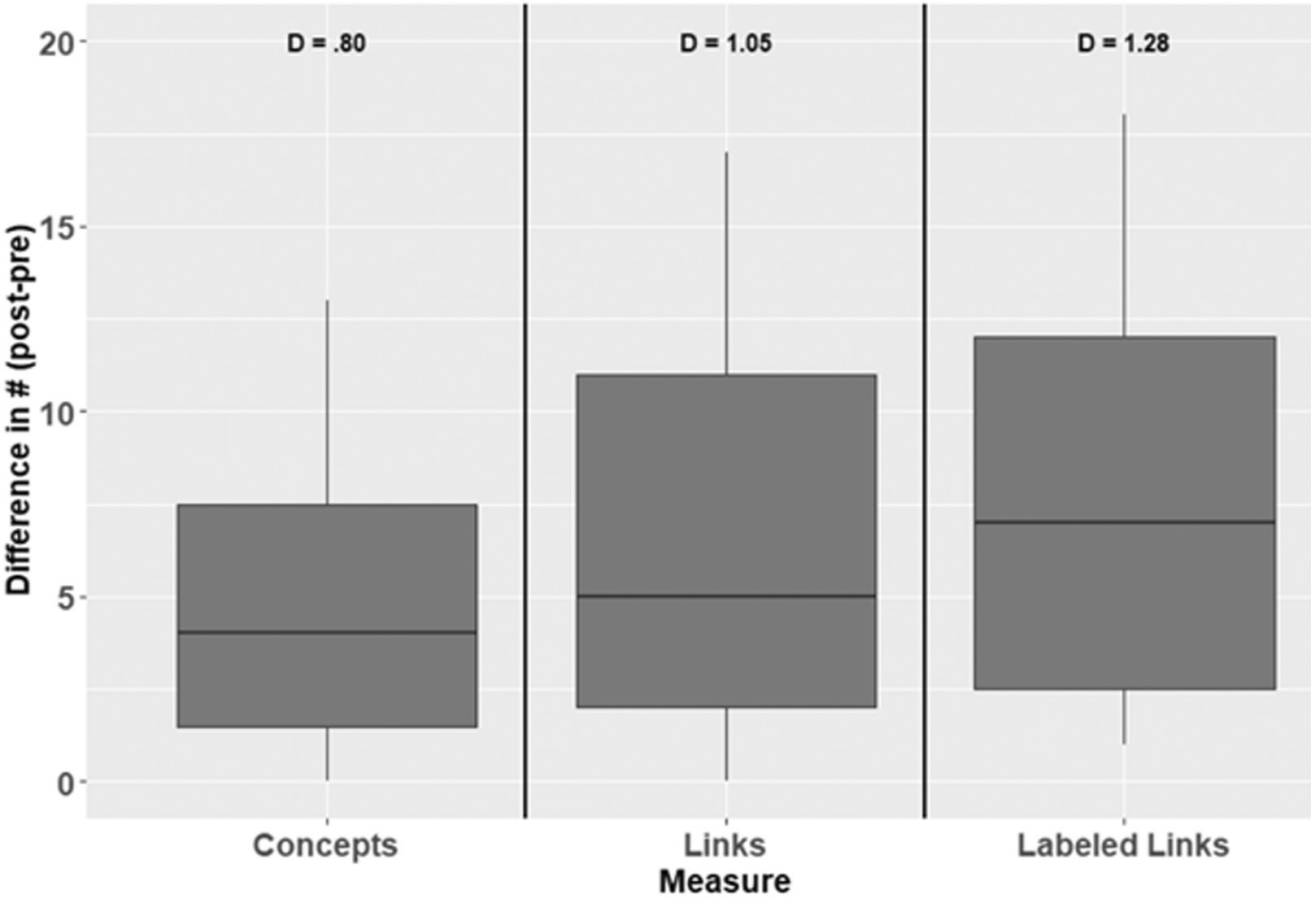
Improvement in concept mapping exercise focused on “biodiversity” from a 2014 implementation of the CREATE method in a Conservation Biology and Biodiversity course at Davidson college. Statistical results are based on within‐subject *t*‐tests, *n* = 28 students who completed the pre‐ and postcourse assessments. Values above each measure are Cohen's *D* effect sizes of the difference in each category between post‐ and precourse concept maps

Students in CBB demonstrated gains in their ability to analyze and synthesize the concept of biodiversity as measured by the total number of concepts (within‐subject *t*‐test *t* = 4.2661, *df* = 27, *p*‐value = .0002), number of links (*t* = 5.5665, *df* = 27, *p*‐value <.0001), and number of labeled links (*t* = 6.7534, *df* = 27, *p*‐value = 2.984e−07) on their concept maps (Figure 7).

### 4.9 Ecology

One of the authors (CP) participated in piloting the assessment tool Eco/Evo MAPS (Measuring Achievement and Progress in STEM) that is aligned with the core concepts outlined by Vision and Change (AAAS, 2011; Summers et al., 2018). The assessment is designed to be administered at different points in the biology curriculum to monitor student progress. In CP's second and third iterations of using CREATE in ecology, students took the assessment at the beginning and end of the semester. The collection of student responses in Ecology was approved by the University of Maine Protection of Human Subjects Review Board IRB# 2015‐06‐07 and the Davidson College HSIRB, protocol #2018‐003.

The assessment has several scenarios, each accompanied by a series of statements. Students rate each statement as likely or unlikely, rather than true or false. In total there are 63 questions, categorized as either Ecological or Evolutionary questions (30 and 33 questions, respectively). Scores are the percentage of the number of statements correctly identified as likely or unlikely. In addition, statements were categorized as assessing one of the five core concepts of Vision and Change (American Association for the Advancement of Science, 2011; Summers et al., 2018). Paired *t*‐tests were applied to pre vs. post for total scores, ecology question scores, and evolution question scores. *t*‐tests and one‐way ANOVAs were used to determine whether there was an effect of having taken AP Biology (t‐test) or an effect of class standing (sophomore, junior, senior) on Eco‐Evo MAPS performance (ANOVA). Data were found to meet the assumptions of these parametric tests.

**FIGURE 8:**
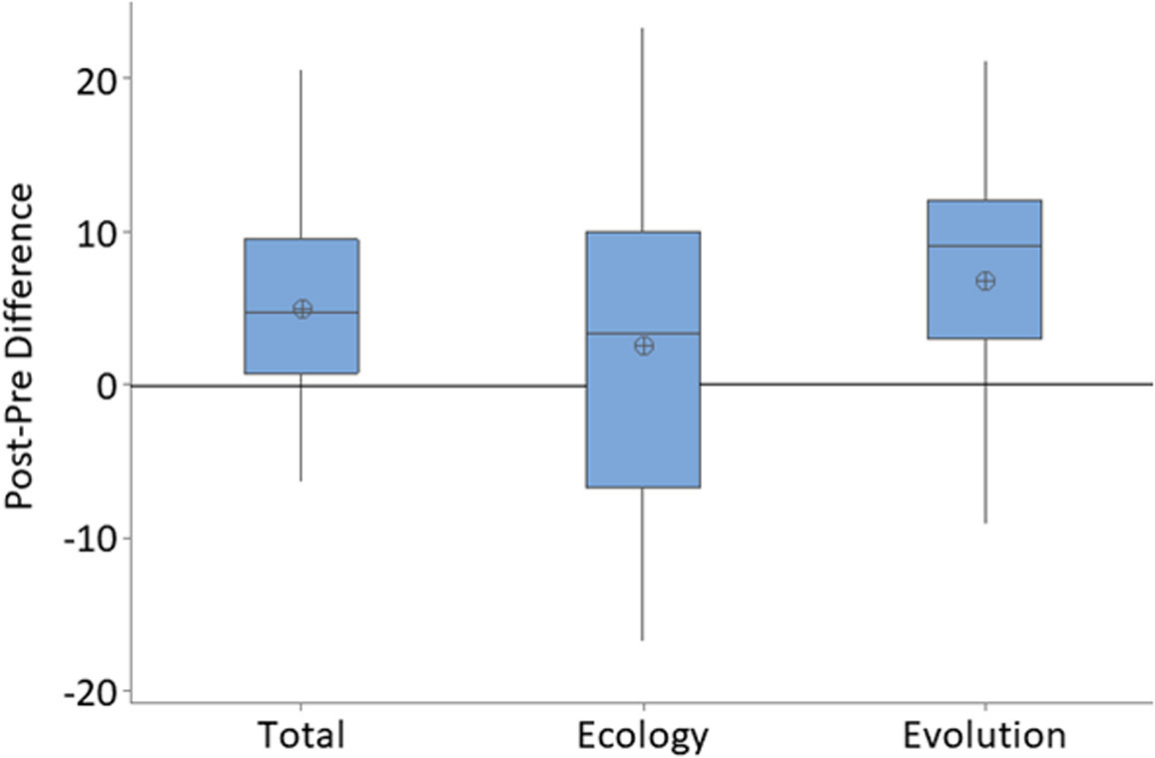
Distribution of eco/Evo MAPS post‐pre score differences pooled across two ecology classes. Total score differential is based on 63 questions, ecology score differential is based on 30 questions, and evolution score differential is based on 33 questions. Line in the middle of the box is the median, the top and bottom of the boxes are the upper and lower quartiles, respectively, and the top and bottom of the whiskers are the maximum and minimum difference in scores, respectively. Symbol near the median is the mean score. *N* = 37

The overall Eco/Evo MAPS score increased significantly during the semester (Figure 8; Table 5). Scores in ecology‐related questions did not increase significantly but scores in evolution‐related questions did (Table 5). We did not find any evidence for an effect of having taken AP Biology in high school nor of class standing on performance either on the precourse or postcourse assessment (Table S1).

**TABLE 5:** Results of paired *t*‐tests for the pre‐ and post‐course eco/Evo MAPS assessment used in an ecology course at Davidson college

	Pre‐course average	Post‐course average	Cohen's *D*	*t*	*p*
2016					
Total	76.98 (2.12)	81.27 (2.26)	0.72	3.21	**.002**
Ecology	79.67 (2.56)	82.50 (2.21)	0.29	1.30	.105
Evolution	72.12 (2.20)	77.73 (2.76)	0.63	2.79	**.006**
2018					
Total	70.12 (2.93)	75.91 (2.60)	0.97	4.00	**.001**
Ecology	74.90 (3.15)	77.25 (2.41)	0.21	0.87	.40
Evolution	64.53 (3.28)	72.73 (3.30)	1.32	5.43	**<.001**

*Note*: *N* = 20 students (2016); *N* = 17 (2018). SE for each average is shown in parentheses. Cohen's *D* measures the effect size.

Bold indicates statistically significant *p*‐values.

## 5 DISCUSSION

We found that students increased their understanding of the scientific process, their attitudes about their own abilities and interest in science, and science practice skills through the CREATE method. Students in CBB demonstrated gains in their ability to analyze and synthesize the concept of biodiversity, and students in Ecology demonstrated increased ecology and evolution content knowledge at the end of the semester.

The novel assessment tool used to assess ecology students (Summers et al., 2018) was designed to provide evidence of student thinking on fundamental ecology and evolution concepts, and especially those with which students often struggle. Scores tended to be higher than those reported by Summers et al. (2018), even in the pretest. This is likely due to students having all completed a year of introductory biology in a biology department with a faculty strongly committed to active learning pedagogies. One half of the year‐long sequence is focused on ecology and evolution concepts. Introductory biology at our institution, as well as HS preparation, may have a greater focus on ecological concepts. Even with a focus on evolutionary concepts and mechanisms, nuances of selection and drift, for instance, may not gel with students until experiencing the concepts again in upper‐level courses, which could explain the larger gains in evolutionary than ecological concepts.

Despite that, we found that overall, students improved in the Eco/Evo MAPS assessment from pre‐ to post‐test. The CREATE pedagogy does not focus on content coverage, so one could hypothesize that content knowledge does not improve in a CREATE ecology course. We found that the CREATE approach improved students' overall ecology and evolution performance but especially in the evolution scores.

Based on assessment results from CBB, students also demonstrated substantial self‐assessed gains in science practice skills and knowledge and attitudes about the scientific process (Table 3 and Figure 6). Although it is somewhat surprising that students did not show similar gains in metacognition, metacognitive skills are very different skills from discipline‐specific skills such as figure annotation, data analysis, and experimental design. Metacognition involves more self‐reflection and changes to “habitats of mind” that may either be less responsive to interventions such as CREATE, or more difficult for students to self‐assess. That said, there is a growing body of literature on methods for encouraging metacognition (Tanner, 2012) and a separate study on the CREATE pedagogy did find student improvements in this learning skill (Kenyon et al., 2016). Results from this study have encouraged KGS to be more deliberate and thoughtful about his promotion of metacognitive skills in his courses since 2018.

Our results indicate that students gain significant content and conceptual knowledge while practicing the process of science on a daily basis, as well as in the assessment of their own abilities. One observation made by CP is that the introductions to papers are filled with concepts and terms that are tangentially related to the topic at hand, and which students investigate and add to concept maps. This allows for a rich discussion of the interrelatedness of ecological concepts and content. The process of repeatedly creating and integrating concept maps fosters deep thinking of terms encountered in an introduction to a paper—students are encouraged to make connections between terms, and this solidifies knowledge of those terms (Beck, 2019). This outcome is seen in the concept map results from CBB (Figure 7), which demonstrate that students make greater gains in their ability to define (label) connections that they do in the simple number of concepts or connections. In other words, students' greatest gains are in the ability to define how ecological concepts are connected, not stating that there is a connection between concepts.

Based on our experiences, student experiences, and student outcomes, we have successfully implemented the CREATE pedagogical approach in these ecologically related courses. The advantages of this approach are that it dispenses with the pedagogical deficiencies inherent to most textbooks in ecology courses and integrates several evidence‐based active learning pedagogies. Furthermore, it is highly flexible, allowing an instructor to adopt part or all the CREATE approach and then adapt the methods to suit the needs and strengths of the instructor. The latter point can be clearly seen in the two case studies, as we have applied the CREATE approach differently in each of our courses. We emphasize different activities, and may not even use all the pedagogies of CREATE, but both courses focus on student‐centered activities that engage students in the process of science and integration of knowledge across disciplines. This facilitates student construction of knowledge of conservation biology and ecology, as well as increased and repeated exposure to science practice skills (Hoskins & Krufka, 2015).

To teach a CREATE course, a teacher must be flexible, often letting the students drive the discussion. While there may be some initial anxiety associated with this, a student‐centered approach benefits students by improving attitudes and performance (Armbruster et al., 2009; Freeman et al., 2014; Hoskins & Krufka, 2015). There is also more planning associated with a CREATE course, to choose papers that relate to the desired topics, are understandable by undergraduate students, and have data visualizations that can be digested and annotated for insights. Placing Units and papers with Units in an order that facilitates student knowledge construction requires forethought as well. The course design challenge may be significant, given all the duties and responsibilities of today's college professor. Ways to overcome this barrier include integrating one or two papers at first, as a supplement to a text, or to begin using concept mapping with concepts discussed in class. The payoff, in our experience, is substantial.

### 5.1 Leveraging CREATE to address DEIJ in ecological courses

Papers can also be chosen to increase the diversity of ways of knowing and representation of underrepresented groups, which textbooks often do not include. As we developed our individual approaches using CREATE, we independently realized that an advantage of this pedagogical approach is that we can select papers that achieve our goal of increasing representation in STEM (Rosemond et al., 2020). This presents opportunities to discuss DEIJ‐related issues that might not happen in a course with a text and a focus on content, including interdisciplinary issues such as social and environmental justice, colonization of science, and non‐European ways of knowing or conducting science (de Vos, 2020; Louis, 2007; Schell et al., 2020; Trisos et al., 2021; Yip, 2021). For instance, reflecting on the relationship between Indigenous science and ecology reveals that both emphasize dynamic, circular, and cyclical processes (Kimmerer, 2012; Salmón, 2000). CREATE also has the potential to increase classroom inclusivity, as the approach includes several principles of inclusive pedagogy, such as collaborative small group work, flexibility, personalization, various ways for students to demonstrate their learning, and a supportive classroom environment (Stevens & Hoskins, 2014). Students construct their own knowledge in a CREATE course, which is an inherently inclusive approach to teaching that naturally fosters a growth mindset. Research on how CREATE affects inclusivity is needed.

### 5.2 Recommendations

The Ecological Society of America has recently unveiled the 4DEE framework for teaching ecology (Berkowitz et al., 2018). The framework consists of core concepts, ecology practice (science practice skills), cross‐cutting themes, and human‐environment interactions. The CREATE approach applied intentionally can address all of these dimensions, with a particular focus on the *process* of science (ecology practice), an approach advocated by Vision and Change. The addition of embedded active learning pedagogies can increase the efficacy of the 4DEE framework (Berkowitz et al., 2018), align courses with the recommendations of Vision and Change (American Association for the Advancement of Science, 2011), and increase student performance (Freeman et al., 2014). We therefore recommend that the teaching of any ecologically related course adopt active learning pedagogies, such as those embedded within CREATE and CREATE itself.

## AUTHOR CONTRIBUTIONS

**Kevin Smith:** Conceptualization (equal); data curation (equal); formal analysis (equal); funding acquisition (lead); investigation (equal); visualization (equal); writing – original draft (equal); writing – review and editing (supporting). **Christopher J Paradise:** Conceptualization (equal); data curation (equal); formal analysis (equal); investigation (equal); visualization (equal); writing – original draft (equal); writing – review and editing (lead).

## CONFLICT OF INTEREST

The authors claim no conflicts of interest.

## Supporting information


Appendix S1

## Data Availability

Assessment data: The data that support the assessment findings of this study are openly available in Dryad at https://doi.org/10.5061/dryad.m37pvmd57 for the Eco/Evo MAPS data.
