# Supplementary material for: Teaching the process of science with primary literature: Using the CREATE pedagogy in ecological courses
Source: Ecol Evol. 2022 Dec 21;12(12):e9644. doi: 10.1002/ece3.9644 (PMC9772399; doi:10.1002/ece3.9644)
Supplement: Supplementary file 1 — Appendix S1 [file ECE3-12-e9644-s001.pdf]

Teaching the Process of Science with Primary Literature: Using the CREATE Pedagogy in Ecological Courses

Kevin G. Smith and Christopher J. Paradise
Departments of Biology and Environmental Studies, Davidson College, Davidson, NC

Supplemental Materials File

Table of Contents

Table of Contents	Page
CREATE Guide forrStudents, from Ecology	2
(note: CP assigns a web reading for a how-to on concept mapping)	2
Explanatory Notes for reading of course syllabi	7
2018 Ecology Syllabus	8
2018 Ecology list of primary literature papers	16
2021 Conservation Biology and Biodiversity readings	19
Example of Redacted Reading	20
Table S1. Results of t-tests for the pre- and post-course Eco/Evo MAPS assessment	27

Learning using CREATE

We will be using a strategy of teaching and learning called the C.R.E.A.T.E. model (Consider Read, Elucidate the hypotheses, Analyze and interpret the data, and Think of the next Experiment). CREATE will actively engage you both in and out of the classroom and will help develop your skills in reading primary scientific literature, critically analyzing data, developing and testing hypotheses, and more.

This teaching strategy clarifies the process of science and makes scientific research more accessible through close analysis of peer-reviewed primary research articles. There will not be a published text for this course, although we will be reading some draft sections of a text I am developing that uses data from the primary literature. In addition, I have provided a link to a Guide to Ecology on Moodle that you can use as a reference. In place of a textbook, we will focus our energies on reading and analyzing papers published in one of four general areas of ecology, plus an overview case study. In the process, you will be challenged to understand and integrate ecological concepts, build your knowledge as the semester progresses, understand methods, interpret data, and propose experiments as next steps. These activities will allow you to experience the creativity of the process of science.

In contrast to other courses that you might have taken here at Davidson, including my own, that have individual students present full papers or individual figures, in CREATE courses all students are challenged to master all figures of each study. The CREATE tools are designed to facilitate your ability to decode research papers before or during class as preparation for in-class detailed analysis. Concept mapping, sketching (cartooning) of experimental designs, and annotation of figures are key tools in the CREATE strategy. These will enable your learning and integration of fundamental ecological concepts, help you visualize connections between ecological concepts, visualize how experiments were done, promote engagement with the data, and prepare you for active discussion.

As the semester progresses, you will compile concept maps, annotated figures, methods sketches, and additional information about experimental techniques or concepts to support your understanding in a book, which you can think of as a portfolio or as your own ecology textbook. Bring your book to every class to work on in class and to serve as a reference.

When you analyze the data, you will be encouraged to think independently about what conclusions you draw rather than to summarize outcomes as stated by the authors. We will form teams and work in these small groups to provide opportunities to work with your peers and experience how different groups may reach different answers or interpretations in response to the same scientific question and results.

Intensive examination of the ecological literature using CREATE can provide you with significant learning gains, transferable analytical skills, and unique insights into ecology. The CREATE strategy requires a high level of preparation and detailed analysis on your part. You may be both surprised and challenged, but the payoff can be immense.

BIO 321 **Dr. Paradise**

Guide to reading the scientific literature in a CREATE course

In the CREATE strategy, you will never see an entire article until you have first read chunks of it. Generally, although not always, you will first download the introduction from Moodle, then the Methods and Results, and then the Discussion. You will be asked to resist the urge to find the article, either online or at the library. Reading the abstract or skipping ahead to the conclusions will detract from the learning experience that has been developed as the CREATE strategy. Here, I give you some generic advice to reading scientific literature.

First, effective readers take notes—it improves recall and comprehension. You may think you'll remember everything you read in researching class assignments, professional papers, proposals, or your thesis, but details slip away. Different sections of scientific papers will be read for several distinct purposes in this course, and thus your note-taking may vary from section to section. For instance, in the introduction you will focus on identifying concepts, linking them together, and integrating them with concepts or terms you learned previously. You may construct a concept map to bring to class, or you and your partners will construct one in class. In this way, you will build your ecological knowledge. In Methods sections, you will focus on translating the written description of experimental design into a sketch, or cartoon.

You will first have access to the introduction

As you read the introduction, write down **every word** that you don't understand. You're going to have to look many of them up, although you can discern many meanings contextually and it will become easier as you build your ecological knowledge. It will be important to keep in mind that you won't understand the paper if you don't understand the vocabulary. Scientific words have precise meanings, as you know by now.

In addition, keep notes on other terms and concepts that you do know. Ultimately, for each introduction, you will build a concept map, linking together known and new concepts and terms. Some terms will be important and fundamental concepts, others will describe methods, processes, taxonomic groups, or environmental factors. In addition to identifying concepts and understanding terms, you will be challenged to read the introduction closely to discern the relationships between these terms. This will allow you to more easily develop a concept map for the introduction and determine the big question of the paper. You should be able to understand why this research has been done in order to explain it to your peers in class.

Identify the specific question(s)

This part may come up in the introduction, or we may get to it after the methods and results are read; it just depends on the structure of the paper and the flow I have developed for the class. For this, try to determine what fundamental ecological question(s) the authors are trying to address with their research. There may be multiple questions or just one. Write them down as part of your note-taking. If it's the kind of research that tests one or more null hypotheses, identify it/them.

Now read the methods section. Draw a diagram for each experiment, showing exactly what the authors did.

What did the researchers do? The CREATE strategy uses cartooning of methods, where you will take the described methods and sketch a picture that represents what the researchers did. We will then compare cartoons to be sure that there is consensus on what was done, how the experiment was

designed, what the controls and sample sizes were, etc. Include as much detail as you need to fully understand the methods and experimental design. You don't need to understand complex analytical methods (such as stable isotope analysis or multivariate statistics), but you should understand what the method was used for and what it is supposed to show. While you won't need to know the methods in enough detail to replicate the experiment, you're not ready to move on to the results until you can explain the basics of the methods to someone else.

Read the results section

Annotation is used to identify components in the figures and tables and to highlight your main points right on the figure or table from the original research. Use the text of the results to help identify the main points. Don't try at this stage to determine what the results mean, just write down the results and point them out in the figures and tables. There may be some challenges at this point if statistical tests are employed that you don't understand. This is one place, among several others, where I will provide some remediation and guidance.

Interpretation occurs before you read the discussion

Using the hypotheses, questions, cartoons, diagrams, and charts and/or graphs, determine what the results mean. Link your interpretations to your concept maps, both current and previous. Analyze your interpretations in light of the experimental methods and goals of the researchers. After all figures and tables have been analyzed, revise your concept map for the paper.

Read the discussion section

Restate what the authors think their results mean. Do your interpretations agree with them? Can you or your peers come up with alternative ways of interpreting the results? Do the authors identify any weaknesses in their own study? Do you see any that the authors missed? What do they propose to do as a next step? Propose your own next steps or next experiments.

Now, go back to the beginning and read the abstract

Does it match what the authors said in the paper? Does it fit with your interpretation of the paper?

A couple of key points to keep in mind

For adequate understanding of an article, you should be prepared to read a section two, three, or even up to four times. You will often be amazed to discover that what seemed completely incomprehensible on the first reading appears to make perfect sense on subsequent readings. You should be comforted to know that even experienced scientists must read articles over and over again. Furthermore, there will be things you simply do not understand because 1) you do not have the adequate background, 2) they are just too complicated, or 3) they simply do not make sense. Do not overlook this last possibility simply because you see something in print.

You should be prepared to do some work in order to acquire sufficient background for adequate understanding of an article. This will include:

1) looking up definitions of words you do not know;
2) looking up points made in references cited by the paper;
3) asking questions of people who may know, including your instructor!

Sketching methods (cartooning) and annotating results

Two key steps in the C.R.E.A.T.E. model (Consider Read, Elucidate the hypotheses, Analyze and interpret the data, and Think of the next Experiment) are *cartooning* and *annotation*, two approaches that will deepen your understanding of the primary literature and the connections between methods, results, and conclusions. This deeper understanding will also increase your understanding of ecological concepts.

Because of inexperience with ecological research methods, even if you read the methods sections you may not understand how studies or experiments are carried out. It is often difficult for undergraduates to visualize what goes on in a research study when reading a methods section. You will be asked to challenge yourself to read the methods closely and to visualize the experimental design on paper.

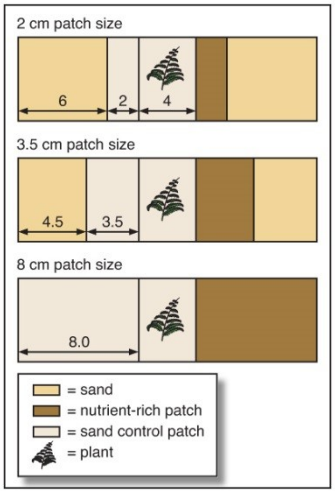

You will be asked to make sketches to represent what goes on in the laboratory or in the field in order to generate the data in particular figures. For many papers we will consider only a subset of the figures and tables so you will visualize the methods only for those results. It could be as simple as an annotated version of a figure that shows the experimental design of a study, as in the figure shown to the right. Annotations for this sketch might include sample sizes, response variables measured (which may actually require an additional cartoon), or species used.

Students often do not like the idea of "cartooning" initially, but it actually provides students with a completely different way to process the information you are reading. Your level of understanding will increase and your frustration will decrease because you have a different outlet to express the ideas presented to you. This is in contrast to reading and rereading and hoping you come to understand it. Cartooning encourages you to more fully understand the techniques and the rationale behind the experiments, the findings, and discover possible shortcomings. Consider this simple example to the right where a cartoon has been made from the directions to baking a cake and how varying any of the factors might lead to alterations in the outcome.

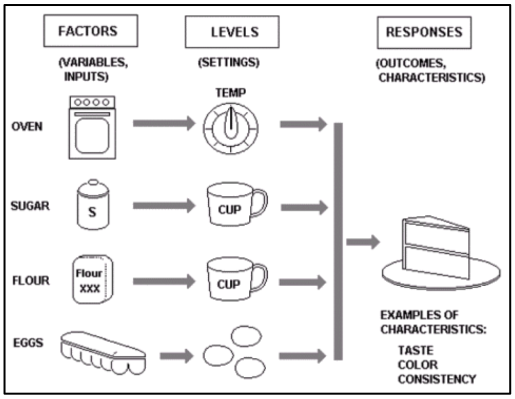

In order to draw something, you have to commit to a belief about what you are representing. That is, students who can bluff their way through a class discussion or successfully rearrange words from a methods section into a flowchart without actually understanding what the words mean are initially stymied when asked to visually represent the methods. Committing to a sketch means

committing to an understanding of something not provided with the paper itself. As you figure out what to draw, you will find yourself going beyond a superficial interaction with the verbiage and begin to develop visualization skills. You will also develop metacognition ability as you define what you do and do not know about the methods. If you realize you can't represent a particular analysis in a sketch because you don't really understand it, then you know you need to look it up or ask your professor.

In class you will work with your groups to create a consensus cartoon or we will work as a class on the board. We must all be in agreement about what was done before we can proceed to determine what the results were and how they should be interpreted.

After cartooning is completed, you will annotate the figures or tables associated with each experiment or study (see below for an example). To do this, rewrite key information from the caption or narrative directly on the figure. This allows you to define what particular arrows are pointing at or what a highlighted box is highlighting or what the axes are or what the treatments or cases represent. In addition, you might have to identify the populations, label of histograms or best-fit curves, what was measured or what is represented in the data.

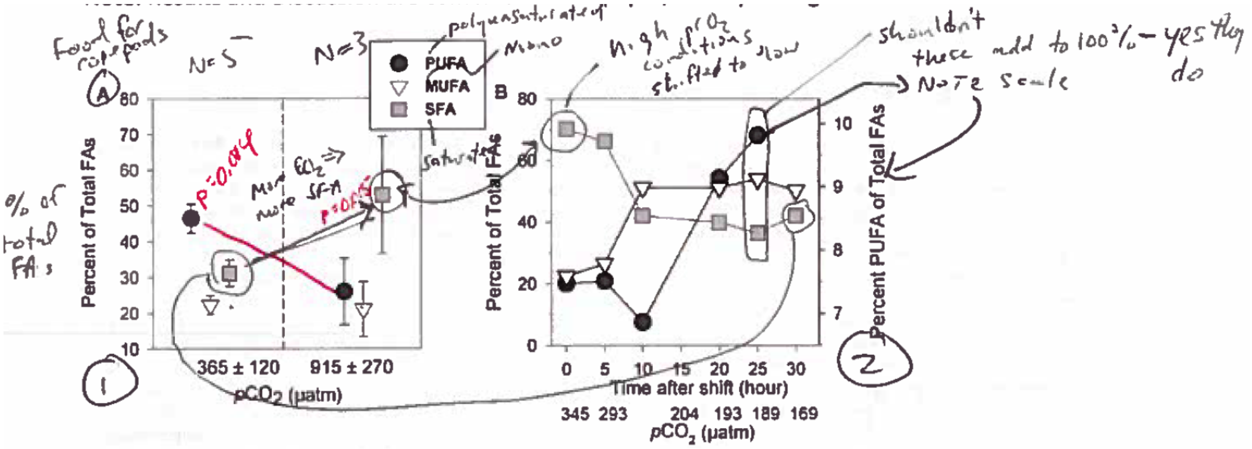

The information needed to identify figure components is present either in captions or in associated narratives. The tracking down of the descriptions of figures and tables helps you build careful reading skills and move beyond cursory skimming of papers. We will sometimes do this in class but you will also be asked to complete this before class so that we have time to discuss and interpret what you have annotated.

The final step in annotation, and the reading of the methods and results, is to retitle the figure and to determine what question or hypothesis was being addressed by the study. This involves paraphrasing the official figure title in your own words. In addition, defining the hypothesis being tested or question being asked helps you see the overall paper in the larger context of the science of ecology.

When you analyze the methods and results on your own, without having access to the conclusions, you will be encouraged to think independently about what conclusions you draw rather than to summarize outcomes as stated by the authors.

The next document is the 2018 syllabus for CP's Ecology course (8 pages). The last half contains the schedule of readings and activities. The readings are all coded by module and paper number. After the syllabus, the papers are decoded and full citations are provided for these papers.

A few notes:

1. The papers used are not static - they change over time, as new, better papers are discovered.

2. Readings are distributed through an LMS, in our case Moodle. Each reading is released prior to its use in class. So for instance, if students are assigned a paper's introduction, they will have access to only that portion of the paper until after the session where it is discussed or used for concept mapping.

3. After the paper is completed (that is, after the session focused on discussion or thinking of the next experiment), the original PDF containing the entire unredacted paper is made available on Moodle, along with the proper citation should students wish to use it for any other assignment.

4. Choice of paper usually boils down to papers that are, in the instructor's estimation, understandable by novices, have decent to excellent data visualizations, are on topics that the instructor wishes the students to consider, or are topics that build on topics discussed earlier.

BIOL 321: Ecology

Meeting times and prerequisites

Class meets on Monday, Wednesday, and Friday, 11:30-12:20, Wall 210

Laboratory meets on or Wednesday 1:30-4:20 (A) or Thursday 1:40-4:20 (B) either in Wall 149 or, if going in the field, at another announced location.

Prerequisite: BIO 112 <u>or</u> BIO 114 <u>or</u> ENV 201 <u>or</u> permission from Dr. Paradise

Course description

Ecology is an interdisciplinary science that examines interactions between organisms and their environment, and the relationship between evolution and these interactions. The interdisciplinary character of ecology allows us to understand nature by placing organisms in the context of their environment. We will explore major concepts and societal concerns in ecology, using case studies, the primary literature, observations, experiments, and the core competencies of science (data analysis, critical thinking, effective communication, quantitative reasoning, and an understanding of the link between science and society).

Bombus citrinus on purple clover

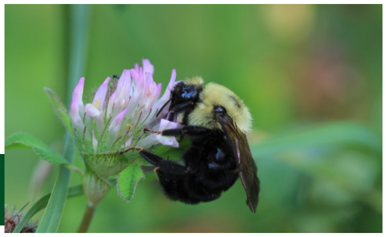

Learning outcomes

Big Picture Learning Outcome: By the end of this course, you should be able to apply ecological theory and concepts and the core competencies of science (data analysis, critical thinking, quantitative reasoning, and effective communication) to explain, relate, evaluate, and hypothesize about ecological phenomena.

Specific Learning Outcomes: After completing this course, you should be able to:

- develop and explain concept maps of ecological terms and concepts, and evaluate concept maps of your peers,
- demonstrate knowledge of the important ecological principles and factors that operate at the levels of the individual organism, the population, the community, and the ecosystem (and describe these levels),
- evaluate and explain how evolutionary processes inform the study of ecology and operate in ecological systems
- describe how organisms' interactions with their environment and other organisms give rise to patterns in abundance and distribution,
- explain how ecological systems change in space and time and compare/contrast changes that occur in different ecological systems,
- evaluate, interpret, apply, and integrate data from the ecological primary literature on individuals, populations, communities and ecological systems,
- formulate research questions, hypotheses, and predictions, and design and propose valid experiments and studies to test hypotheses in ecology,
- demonstrate proficiency with common approaches for quantitative analysis and graphical representation of ecological data and models, and
- apply ecological concepts to current environmental challenges, including global climate change.

BIO 321: Ecology

Instructor:

> Dr. Chris Paradise
> Wall 192 or 146 (Lab)
> Phone: 2890
> e-mail: chparadise @ davidson.edu
> Office hours: Mon 9:30-10:20; 1:30-2:20; Wed 10:30-11:20, Thur 9:40-10:55, Fri. 1:30-2:20, or by appt.

Syllabus components:

Reading Assignments	2
Communication & Tech	2
Assignments, etc.	2
Attendance	3
Honor code	4
Evaluation	4
Accessibility	4
Support	5
Class schedule	5-7
Lab/field	7-8

Reading Assignments

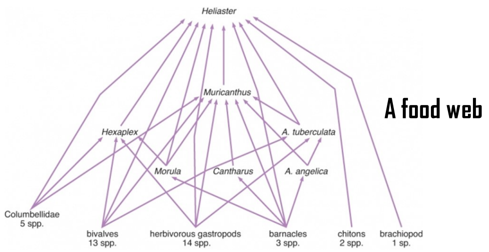

A food web

There is no ecology textbook in this course. We will read research papers from the primary literature that will allow us to launch into a deeper investigation of ecological terms, concepts, and data.

Assignments, discussion, participation

Writing assignments

Ecology Portfolio: You will create a portfolio in which you will keep assigned readings and accompanying assignments (detailed below). This portfolio will be collected 2 times during the semester and evaluated based on the quality and quantity of thought and effort. I will also check recent work and give you feedback, often in class.

The materials in your portfolio should be created by you alone , even if you worked with others during in-class exercises or in a study group outside of class. Directly copying portfolio materials from another student is a violation of the Honor Code. Your portfolio should reflect your own thoughts, style, and effort. Bring this to class with you every day.

You will create some or all of the following for each paper that we read:

- Concept maps: Concept maps are graphical tools for organizing and representing knowledge. They include concepts, enclosed in circles or boxes, with relationships indicated by a connecting line linking concepts. Words on the line, referred to as linking phrases, specify the relationship.
- Cartoons of methods: Cartooning is a way of representing the methods. Your challenge is to sketch the methods so that they accurately reflect what the researchers did.
- Figure and Table annotations: Annotating figures is a great way to familiarize yourself with and summarize the results. Annotation is used to identify components in the figures and tables and to highlight main points right on the figure or table from the original research.
- Defining of questions or hypotheses: Summarizing the main questions, in your own words, is a powerful way to show that you understand the fundamental questions or hypotheses.
- Analysis and interpretation of results: Determine the logic of each experiment, examine the correspondence between experimental design and results, interpret the significance of the data, and evaluate and criticize the authors' interpretations.
- Listing of key points: Make a list of the key points from the study, which can be drawn from the hypotheses, the annotations of figures and tables, and interpretation of results.
- Defining/describing the next experiment: The final challenge is to think creatively about what you would do next if you were the researcher.

Integrative Essays: Several written assignments will be used to assess your ability to integrate course material and concepts. These will be 2-3 page essays on topics related to papers we read and discuss in class, with the addition of other primary literature you research, analyze, and integrate.

BIO 321: Ecology

The distribution and abundance of organisms

Communication and technology

Course announcements, reminders, and ecology-in-the-news messages will be conveyed via e-mail and/or Moodle. This course uses Moodle for assignments and readings. **Please be responsible in your use of paper and printing. I encourage you to read assignments electronically whenever possible; but bring your notes or devices to class!**

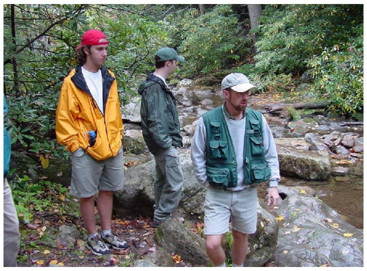

In the field, studying stream insect diversity, Fall 2001

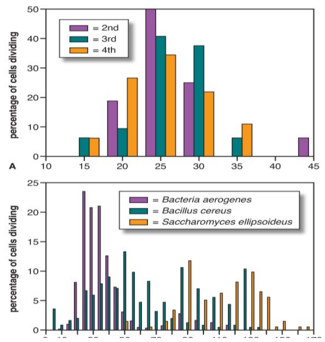

Population growth dynamics

Assignments, discussion, participation, cont'd

Exams

There will be three exams, one of which may be an oral exam. Dates are shown in the semester schedule. I will provide you with additional details and examples ahead of time. The first exam will be written, the second one will be an oral exam, and the final exam will be written.

Daily assignments

You will find it convenient and helpful to meet with your peers to discuss assignments and readings. Keep in mind that your written work must be your own and should reflect your own understanding of the assignment. Perhaps a good approach to take is to read the assignment on your own, then discuss it with teammates, and then complete the assignment on your own. The assignments will be steps toward the content that you are building for your book. Often, I will expect you to show up with the work, and in other situations I'll ask you to produce something during class. Most of these items will eventually be incorporated in your book.

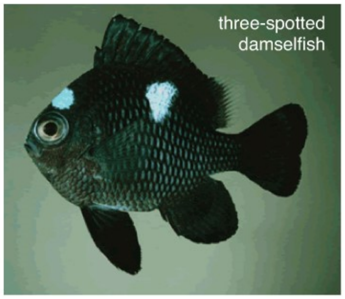

Three-spotted damselfish, *Pomacentrus tripunctatus*

Class

We will frequently have small group and class discussions on the readings. I will call on volunteers or select non-volunteers to summarize the material, relate it to the material we are currently covering, relate it to the themes of the course, or answer any number of other questions. Your preparation for class on these days will count for class participation. In addition, we will often have short discussions regarding the textbook reading; this will encourage you to read ahead.

Be prepared for class, whether the format for that day will be group work, discussion, field trip, or laboratory exercise. Material covered in lecture will supplement the text, and understanding the lecture will depend upon your reading before class. Unavoidable absences may occur, and in such cases, will require a legitimate excuse. There will be many out-of-class assignments, the completion of which will be critical to your participation in class. If you don't do the assigned out-of-work class, you will not be able to effectively participate in class.

Lab assignments

There will be three short laboratory reports, written individually but based on group field/lab work. We will form groups using an online assessment tool called CATME – I will provide further instructions via e-mail. See manual for more information.

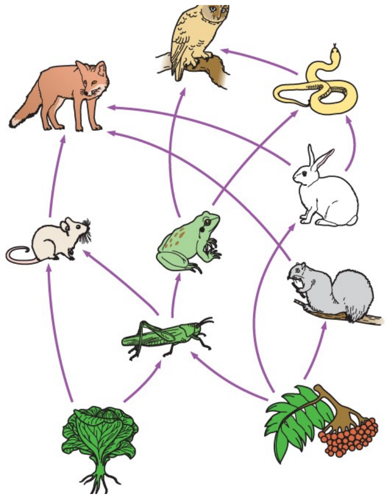

An example of a simple food web

Attendance

Tardiness is not acceptable. I start on time, and I expect you to be in class or at a meeting place when I begin; I will not wait for anyone for field trips. It's distracting and disrespectful to come in late. If you are going to be absent or late and think you have a valid excuse, see or e-mail me. This applies to assignments; any assignment not turned in on time, and without a valid excuse, will receive an automatic 10% deduction, with another 10% for each additional day it is late. Consistent absences, tardiness, non-participation, and unpreparedness will affect your participation/attendance grade. You will not be graded on attendance per se, but I will always be taking notes on class participation and the quality of your in- and out-of-class work. In short, if you are not present, you will not receive credit for participation or the miscellaneous homework/in-class assignments, and the quality of your personal portfolio will suffer.

Honor code

You will pledge all assignments in this course under the Honor Code. For group lab work, collaboration on results (figures, tables, and statistics) is allowed. For writing assignments, it is important to understand and be aware of proper citation. See the statement on plagiarism at http://www.bio.davidson.edu/dept/plagiarism.html. I will provide instructions for citations and references in documents posted on Moodle.

Accessibility statement

I am committed to accommodating students with learning or physical disabilities. Your success in this class is important to me. If there are circumstances that may affect your performance in this class, please let me know as soon as possible so that we can work together to develop strategies for adapting assignments to meet both your needs and the course requirements.

Davidson College values the diversity of its community and is an equal access institution that admits otherwise qualified applicants without regard to disability. The college will review requests for accommodations related to disability and will grant those that are determined to be reasonable and maintain the integrity of a program or curriculum. To make such a request or to begin a conversation about a possible request, please contact the Office of Academic Access and Disability Resources, which is located in the Center for Teaching and Learning in the E.H. Little Library: Beth Bleil, Director, bebleil@davidson.edu, 704-894-2129; or Alysen Beaty, Assistant Director, albeaty@davidson.edu, 704-894-2939. It is best to submit accommodation requests within the drop/add period; however, requests can be made at any time in the semester. Please keep in mind that accommodations are not retroactive.

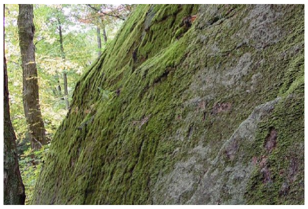

Moss covered rock

People learn in different ways and a course may not accommodate each student's needs. For example, you may prefer to process information by speaking and listening, so that some of the written handouts I provide may be difficult to absorb. Please talk to me as soon as you can about your learning needs and how this course can best accommodate them. If you do not have a documented disability, remember that other support services (Writing Center, Speaking Center and Math and Science Center) are available to all.

I am committed to accommodating and working with students from all backgrounds. As with students with disabilities, if there are circumstances that may affect your performance, please let me know as soon as possible so that we can work together to develop strategies for adapting assignments to meet both your needs and the requirements of the course.

BIO 321: Ecology

The distribution and abundance of organisms

Evaluation

Grades will be assigned using the following scale. If you are unsure about your standing, please contact me:

A:	93.0-100%
A-:	90.0-92.9%
B+:	87-89.9%
B:	83.0-86.9%
B-:	80.0-82.9%
C+:	77-79.9%
C:	73.0-76.9%
C-:	70.0-72.9%
D+:	65-69.9%
D:	60.0-64.9%
F:	< 60%

Grade distribution

Individually constructed portfolio: could include all or some of the following:<br>Concept maps, cartooning methods and annotating<br>Defining questions or hypotheses<br>Analysis and interpretation of results<br>Listing of key points<br>Defining/describing the next experiment	100
Exams (3 @ 100 points each)	300
Integrative Essays (3 @ 70, 90, 90 points, first to last)	250
Laboratory (3 individually-written reports @ 70, 80, 100	250
Participation, preparation, & attendance	100
Total	1000

Campus support for your learning

BIO 321: Ecology

The distribution and abundance of organisms

The Math & Science Center (MSC) offers free assistance to students in all areas of math and science, with a focus on the introductory courses. Trained and qualified peers hold one-on-one and small-group tutoring sessions on a drop-in basis or by appointment, as well as recap sessions ahead of reviews. Emphasis is placed on thinking critically, understanding concepts, making connections, and communicating effectively. In addition, students can start or join a study group and use the MSC as a group or individual study space. Located in the Center for Teaching & Learning (CTL) on the 1st floor of the Library, drop-in hours are usually Sunday through Thursday, 8-11 PM, but I will announce the schedule as soon as it comes out. Appointments are available. For more info, visit http://www3.davidson.edu/cms/x39569.xml.

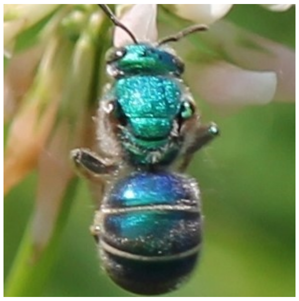

Halictid bee

The Speaking Center @ Davidson College offers the services of trained student tutors to support speaking across the curriculum. At any point of the process, from selecting a topic to delivering the speech, the Center can assist you in learning to speak, and speaking to learn. No appointment is necessary. Located in Chambers B39 (north basement), the Speaking Center includes private rooms, a camera and playback equipment, and resources to help students collaborate with tutors. Students wishing to keep copies of their presentations can bring recordable DVDs. The Writing Center is located near Studio D and the Center for Teaching and Learning in the Library. Hours for the Speaking and Writing Centers will be announced in class.

Tentative Schedule

Date	Topic	Before class	During class
1/17	Forming the learning community; concept mapping; What is ecology?	"Ecology: A short introduction"	Form learning community; discuss CREATE learning model; concept mapping
1/19	**Module 1: Global change ecology:** Do organisms evolve when global climate changes?	M1P1 Introduction	Concept mapping in class
1/22	Do organisms evolve when global climate changes?	M1P1 Methods and Results; sketch cartoon of methods	Annotate results for M1P1; interpret, develop key points
1/24	Do organisms evolve when global climate changes?<br><br>Does timing of biological events change during global climate change?	M1P1 Discussion<br><br>M1P2 Introduction; concept mapping	Revise concept map; design next experiment for M1P1<br><br>Discuss concept maps; read and cartoon methods for M1P2
1/26	Does timing of biological events change during global climate change?	M1P2 results; begin annotation	M1P2 interpretation, integration
1/29	Does timing of biological events change during global climate change?	M1P2 Discussion	Key points and design next experiment
1/31	**Module 2: Ecosystems:** What is a system?	M2P1 Introduction & Methods; create concept map	Cartoon methods; annotate and interpret results
2/2	What factors affect primary productivity?	M2P2 Introduction; create ecosystem energetics concept map	Discuss concept maps; M2P2 Methods: cartoon methods
2/5	What factors affect primary productivity?	M2P2 Results	Annotate results; interpretation, integration;
2/7	What factors affect primary productivity?	M2P2 Discussion	Discuss interpretation; devise next steps; revise concept map
2/9	How do nutrients cycle in ecosystems?	M2P3 Introduction	Create nutrient cycle concept map; **Collect Portfolios**
2/12	How do nutrients cycle in ecosystems?	M2P3 Methods; cartoon methods	Review methods, annotate and interpret results; revise concept map

Tentative Schedule

Date	Topic	Before class	During class
2/14	How do nutrients cycle in ecosystems?	M2P3 Discussion	Interpretation, integration, and next steps
2/16	What is an anthrome? **Exam #1 distributed**	M2P4 Introduction; create concept map	M2P4 Methods and Results; annotate results and interpret
2/19	What is an anthrome?	M2P4 Discussion	Revise interpretation and integrate
2/21	**Module 3: Individuals:** What adaptations do organisms have to obtain resources?	M3P1 Introduction	Create adaptation concept map
2/23	What adaptations do organisms have to obtain resources?	M3P1 Methods	Cartoon methods; read, annotate and interpret results
2/26	What adaptations do organisms have to obtain resources?	M3P1 Discussion	Discuss interpretation for M3P1; concept map of the niche; **Essay #1 DUE**
2/28	How does selection of habitat affect an individual's success?	M3P2 Introduction; add to adaptation concept map	M3P2 Methods: cartoon methods
3/2	How does selection of habitat affect an individual's success?	M3P2 Results	Annotate results and interpret; **Forest report DUE**
3/5-9	**NO CLASS; Fall Break**		
3/12	How does selection of habitat affect an individual's success?	M3P2 Discussion	Interpretation, integration, and next steps
3/14	Does disturbance affect the evolution of life history strategies?	M3P3 Introduction	Create life history concept map
3/16	Does disturbance affect the evolution of life history strategies?	M3P3 Methods & Results	Cartoon methods, annotate and interpret results; revise concept map
3/19	NO CLASS; CP out of town		
3/21	Does disturbance affect the evolution of life history strategies?	M3P3 Discussion	Discuss interpretation and integrate; design next experiments; **Essay #2 DUE**
3/23	**Module 4: Populations:** How do biotic factors affect population dynamics?	M4P1 Introduction	Create population growth and dynamics concept map
3/26	How do biotic factors affect population dynamics?	M4P1 Methods and Results; cartoon methods	Annotate and interpret results
3/28	How do biotic factors affect population dynamics? **Schedule oral exams**	M4P1 Discussion	Discuss interpretation and integrate; revise concept map
3/30	What is density-dependence and how does it affect populations?	M4P2 Introduction	Create density-dependence concept map
4/2	NO CLASS; Easter Break		
4/4	What is density-dependence and how does it affect populations?	M4P2 Methods and Results	Cartoon methods, annotate and interpret results
4/6	What is density-dependence and how does it affect populations?	M4P2 Discussion; read also Density-Independence	Discuss interpretation and integrate; revise concept map
4/9	How do metapopulations vary spatially and temporally?	M4P3 Introduction; create concept map	Cartoon methods, annotate and interpret results
4/11	How do metapopulations vary spatially and temporally?	M4P3 Results & Discussion; Annotate results	Discuss interpretation and integrate; revise concept map

Tentative Schedule

Date	Topic	Before class	During class
4/13	**Module 5: Communities:** Is there a relationship between competition and the niche?	M5P1 Introduction	Create community ecology concept map
4/16	Is there a relationship between competition and the niche?	M5P1 Methods and Results	Cartoon methods, annotate and interpret results; **Collect Portfolios**
4/18	Is there a relationship between competition and the niche?	M5P1 Discussion	Discuss interpretation and integrate; revise concept map; **Big Data Report DUE**
4/20	What factors affects food chain length in communities?	M5P2 Introduction and Methods (Food Web Ch.)	Add to community ecology concept map; cartoon methods
4/23	What factors affects food chain length in communities?	M5P2 Results and Discussion	Annotate and interpret results; integrate knowledge
4/25	How is global climate change affecting our oceans?	M5P3 Introduction (Food Web Ch.)	Concept mapping for global change ecology and food webs—integrate
4/27	How is global climate change affecting our oceans?	M5P3 Methods and Results	Cartoon methods and annotate results; interpret, revise concept map; **Essay #3 DUE**
4/30	How is global climate change affecting our oceans?	M5P3 Discussion	Discuss interpretation; revisit GCC module
5/2	Does habitat loss lead to biodiversity loss?	M5P4 Introduction	Create concept map;
5/4	Does habitat loss lead to biodiversity loss?	M5P4 Methods, Results	Annotate and interpret results
5/7	Does habitat loss lead to biodiversity loss? **Exam #3 distributed**	M5P4 Discussion	Discuss interpretation; integrate knowledge; design experiments
5/9	NO CLASS; Attend Alenda Lux		**Pond Report DUE**

Ecology Laboratory and Field Information

MATERIALS: Laboratory & Field Exercises available in MOODLE; proper clothes for field (shoes, not sandals; hat; sunblock, etc.)

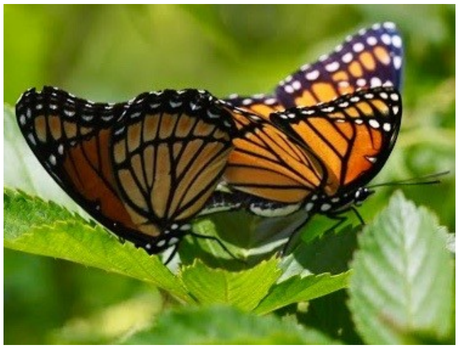

Monarch butterflies mating

ASSIGNMENTS, PARTICIPATION & EVALUATION

Evaluation: The laboratory portion of your grade will be based on a total of 250 points to be distributed as follows; 1) one individual report on forest succession (70 points), 2) one individual report on ecological big data (90 points), 3) one individual report on pond community ecology (90 points), and 4) participation and attendance (included in overall P&A).

Tentative Lab/Field Schedule

Dates	Exercise	Notes
1/17, 18	Safety in the laboratory and field, overview of laboratory portion of course; creating teams; Visit greenhouse and farm	Sign laboratory and field agreements<br>Read Sections 1 and 2<br>Dress for the field
1/24, 25	Forest community ecology data collection	Read Section 3<br>Dress for field work
1/31, 2/1	Forest community ecology data collection	Read Section 3<br>Dress for field work
2/7, 8	Forest community ecology data analysis<br>Design project and protocol using pond mesocosms	Analyze data and discuss w/ instructor; Read Sections 4 and 6; lab report #1 assigned;
2/14, 15	Forest community ecology data analysis; discuss writing scientific reports; set up pond mesocosms	Analyze data and discuss w/ instructor; Refer to Sections 3, 4 & 6; Report #1 assigned;
2/21, 22	Check pond mesocosms; begin big data study	Read Sections 5<br>Dress for field work
2/28, 3/1	Add zooplankton to pond mesocosms; Continue big data study	Refer to Sections 4 & 5<br>Dress for field work<br>**Forest community ecology report DUE**
3/7, 8	Spring break	NO lab this week
3/14, 15	Sample pond mesocosms; add tadpoles; continue work on big data study	Dress for field work
3/21, 22	Continue work on big data study	
3/28, 29	Analyze big data study and construct results section	Begin writing big data ecology report
4/4, 5	NO scheduled lab—Oral exams this week	Continue writing big data ecology report
4/11, 12	Wrap up pond mesocosm study; clean tanks, and process and analyze data	Begin writing up pond community ecology report
4/18, 19	Analyze pond mesocosm study data	**Big data ecology report due**
4/25, 26	Work on pond mesocosm report	Continue working on pond results and report
5/2, 3	Work on pond mesocosm report	**Course evaluations; course wrap-up**
5/8	Last day of class	**Pond mesocosm report due**

2018 Ecology list of primary literature papers

Topic	321	CITATION
What is ecology?		Paradise CJ. 2018. Introduction to ecology.
**Module 1: Global change:** Do organisms evolve when global climate changes?	M1P1	Franks SJ, Sim S, Weis AE: Rapid evolution of flowering time by an annual plant in response to a climate fluctuation, *Proc Natl Acad Sci USA* 104(4):1278-1282, 2007.
Does timing of biological events change during global climate change?	M1P2	Bradley, Nina L., A. Carl Leopold, John Ross, and Wellington Huffaker. 1999. "Phenological Changes Reflect Climate Change in Wisconsin." Proceedings of the National Academy of Sciences of the United States of America 96 (17): 9701–4.
**Module 2: Ecosystems:** What is a system?	M2P1	Forbes, S.A. 1887. The lake as a microcosm. Bull. Sci. Assoc., Peoria, Illinois, pp 77–87. Reprinted in Illinois Nat. Hist. Survey Bulletin 15(9):537–550.
What factors affect primary productivity?	M2P2	Ponce Campos G et al. 2013. "Ecosystem resilience despite large-scale altered hydroclimatic conditions" Nature 494:349-353
How do nutrients cycle in ecosystems?	M2P3	Bormann et al. 1974. The Export of Nutrients and Recovery of Stable Conditions Following Deforestation at Hubbard Brook. Ecological Monographs 44(3):255-277. http://www.jstor.org/stable/2937031
What is an anthrome?	M2P4	Ellis EC & Ramankutty N. 2008. Putting people in the map: anthropogenic biomes of the world. Frontiers in Ecology and Environment 6(8): 439–447, doi: 10.1890/070062
**Module 3: Individuals:** What adaptations do organisms have to obtain resources?	M3P1	Chittka L, Ings TC, Raine NE. 2004. Chance and adaptation in the evolution of island bumblebee behavior. Population Ecology 46:243-251. DOI: 10.1007/s10144-004-0180-1
How does selection of habitat affect an individual's success?	M3P1	Benson JF & Chamberlain MJ. 2007. Space Use and Habitat Selection by Female Louisiana Black Bears in the Tensas River Basin of Louisiana. Journal of Wildlife Management 71(1):117-126. DOI: 10.2193.2005-580
Does disturbance affect the evolution of life history strategies?	M3P3	Shryrock DF, DeFalco LA, Esque TC. 2014. Life-history traits predict perennial species response to fire in a desert ecosystem. Ecology and Evolution 4(15): 3046–3059. doi: 10.1002/ece3.1159
**Module 4: Populations:** How do biotic factors affect population dynamics?	M4P1	Hoy, Sarah R., Steve J. Petty, Alexandre Millon, D. Philip Whitfield, Michael Marquiss, Martin Davison, and Xavier Lambin. 2015. "Age and Sex-Selective Predation Moderate the Overall Impact of Predators." Journal of Animal Ecology 84 (3): 692–701. doi:10.1111/1365-2656.12310.
		Knight, Tiffany M. 2007. "Population-Level Consequences of Herbivory Timing in Trillium grandiflorum." American Midland Naturalist 157 (1): 27–38.
What is density-dependence and how does it affect populations?	M4P2	Rodel HG, Bora A, Kaiser J, Kaetzke P, Khaschei M, von Holst D. 2004. Density-dependent reproduction in the European rabbit: a consequence of individual response and age-dependent reproductive performance. Oikos 104:529-539.
		Unnsteinsdottir ER, Hersteinsson P. 2009. Surviving north of the natural range: the importance of density independence in determining population size. Journal of Zoology 277:232–240.
How do metapopulations vary spatially and temporally?	M4P3	Husband BC & Barrett SCH. 1998. Spatial and Temporal Variation in Population Size of Eichhornia paniculata in Ephemeral Habitats: Implications for Metapopulation Dynamics. Journal of Ecology 86(6):1021-1031. Stable URL: http://www.jstor.org/stable/2648665
**Module 5: Communities:** Is there a relationship between competition and the niche?	M5P1	Bolnick DI et al. 2010. Ecological release from interspecific competition leads to decoupled changes in population and individual niche width. Proceedings of the Royal Society B. doi:10.1098/rspb.2010.0018
What factors affects food chain length in communities?	M5P2	Post DM, Pace ML, Hairston, Jr. NG. 2000. Ecosystem size determines food-chain length in lakes. Nature 405:1047-1049.
How is global climate change affecting our oceans?	M5P3	Rossoll D, Bermúdez R, Hauss H, Schulz KG, Riebesell U, Sommer U, & Winder M. 2012. "Ocean Acidification-Induced Food Quality Deterioration Constrains Trophic Transfer." PLOS 7 (4): e34737. doi:10.1371/journal.pone.0034737.

Does habitat loss lead to biodiversity loss?	M5P4	Puttker T, de Arruda Bueno A, Prado PI, Pardini R. 2015. Ecological filtering or random extinction? Beta-diversity patterns and the importance of niche-based and neutral processes following habitat loss. Oikos 124:206-215. doi: 10.1111/oik.01018

Citations, which are added to the Learning Management System after paper discussion is complete

M1P1: Franks SJ, Sim S, Weis AE. 2007. Rapid evolution of flowering time by an annual plant in response to a climate fluctuation, *Proc Natl Acad Sci USA* 104(4):1278-1282.

M2P2: Bradley NL, Leopold AC, Ross J, Huffaker W. 1999. Phenological changes reflect climate change in Wisconsin, *Proc Natl Acad Sci USA* 96(17):9701–4.

M2P1: Forbes SA. 1887. The lake as a microcosm. Bull. Sci. Assoc., Peoria, Illinois, pp 77–87. Reprinted in Illinois Nat. Hist. Survey Bulletin 15(9):537–550.

M2P2: Ponce Campos GE, Moran MS, Huete A, et al. 2013. Ecosystem resilience despite large-scale altered hydroclimatic conditions. Nature 494:349-353. doi:10.1038/nature11836.

M2P3: Bormann F, Likens GE, Siccama TG, Pierce RS, Eaton JS. 1974. The export of nutrients and recovery of stable conditions following deforestation at Hubbard Brook. Ecological Monographs 44(3):255-277. http://www.jstor.org/stable/2937031

M2P4: Ellis EC, Goldewijk KK, Siebert S, Lightman D, Ramankutty N. 2010. Anthropogenic transformation of the biomes, 1700 to 2000. Global Ecology and Biogeography 19(5):589–606. doi:10.1111/j.1466-8238.2010.00540.x. and Ellis EC, Ramankutty N. 2008. Putting people in the map: Anthropogenic biomes of the world. Frontiers in Ecology and the Environment 6(8):439–47. doi:10.1890/070062.

M3P1: Chittka L, Ings TC, Raine NE. 2004. Chance and adaptation in the evolution of island bumblebee behavior. Population Ecology 46:243-251. DOI: 10.1007/s10144-004-0180-1

M3P2: Benson JF & Chamberlain MJ. 2007. Space use and habitat selection by female Louisiana black bears in the Tensas River Basin of Louisiana. Journal of Wildlife Management 71(1):117-126. DOI: 10.2193.2005-580

M3P3: Shryrock DF, DeFalco LA, Esque TC. 2014. Life-history traits predict perennial species response to fire in a desert ecosystem. Ecology and Evolution 4(15): 3046–3059. doi: 10.1002/ece3.1159

M4P1a: Hoy SR, Petty SJ, Millon A, Whitfield DP, Marquiss M, Davison M, Lambin X. 2015. Age and sex-selective predation moderate the overall impact of predators. Journal of Animal Ecology 84 (3): 692–701. doi:10.1111/1365-2656.12310.

M4P1b: Knight TM. 2007. Population-level consequences of herbivory timing in *Trillium grandiflorum*. American Midland Naturalist 157 (1): 27–38.

M4P2a: Rodel HG, Bora A, Kaiser J, Kaetzke P, Khaschei M, von Holst D. 2004. Density-dependent reproduction in the European rabbit: a consequence of individual response and age-dependent reproductive performance. Oikos 104:529-539.

M4P2b: Unnsteinsdottir ER, Hersteinsson P. 2009. Surviving north of the natural range: the importance of density independence in determining population size. Journal of Zoology 277:232–240.

M4P3: Husband BC, Barrett SCH. 1998. Spatial and temporal variation in population size of *Eichhornia paniculata* in ephemeral habitats: Implications for metapopulation dynamics. Journal of Ecology 86(6):1021-1031. Stable URL: http://www.jstor.org/stable/2648665

M5P1: Bolnick DI, Ingram T, Stutz WE, Snowberg LK, Lau OL, Paull JS. 2010. Ecological release from interspecific competition leads to decoupled changes in population and individual niche width. Proceedings of the Royal Society B. doi:10.1098/rspb.2010.0018

M5P2: Post DM, Pace ML, Hairston, Jr. NG. 2000. Ecosystem size determines food-chain length in lakes. Nature 405:1047-1049.

M5P3: Rossoll D, Bermúdez R, Hauss H, Schulz KG, Riebesell U, Sommer U, & Winder M. 2012. Ocean Acidification-Induced Food Quality Deterioration Constrains Trophic Transfer. PLOS 7 (4): e34737. doi:10.1371/journal.pone.0034737.

M5P4: Puttker T, de Arruda Bueno A, Prado PI, Pardini R. 2015. Ecological filtering or random extinction? Beta-diversity patterns and the importance of niche-based and neutral processes following habitat loss. Oikos 124:206-215. doi: 10.1111/oik.01018

Summary of the papers used in a recent (2021) version of CBB using the CREATE method.

**Module**	**Papers**
Module 1: What is Nature? What is Conservation Biology/Conservation Science?	M1P1: Ducarmé, F. and D. Couvet. 2020. What does 'nature' mean? Palgrave Communications 6-14<br>M1P2: Kareiva, P. and M. Marvier. 2012. What is conservation science? BioScience 62: 962-969<br>M1P3: Salmón, E. 2000. Kincentric ecology: Indigenous perspectives of the human-nature relationship. Ecological Applications 10:1327-1332<br>M1P4: Kimmerer, R.W. 2012. Searching for synergy: Integrating traditional and scientific ecological knowledge in environmental science education. Journal of Environmental Studies and Sciences 2:317-323
Module 2: What is biodiversity? How and why does it vary around the world?	M2P1: Brown, J.H. 2014. Why are there so many species in the tropics? Journal of Biogeography 41:8-22.<br>M2P2: Kerr, J.T. and L. Packer. 1997. Habitat heterogeneity as a determinant of mammal species richness in high-energy regions. Nature 385:252-254
Module 3: What does biodiversity *do*?	M3P1: Ostfeld, R.S. and F. Keesing. 2000. Biodiversity and disease risk: the case of Lyme disease. Conservation Biology 14:722-728.<br>M3P2: Halsey, S. 2019. Defuse the dilution effect debate. Nature Ecology & Evolution 3:145-146.<br>M3P3: Hanski, I, et al. 2012. Environmental biodiversity, human microbiota and allergy are interrelated. PNAS 109:8334-8339.<br>M3P4: Crutsinger, G.M. et al. 2006. Plant genotypic diversity predicts community structure and governs and ecosystem process. Science 313:966-968.
Module 4: What is happening to biodiversity? Why and how?	M4P1: Brooks, T.M. et al. 2002. Habitat loss and extinction in the hotspots of biodiversity. Conservation Biology 16:909-923.<br>M4P2: McClenachan, L. 2009. Documenting loss of large trophy fish from the Florida Keys with historical photographs. Conservation Biology 23:636-643.<br>M4P3: Hughes, T.P. et al. 2007. Phase shifts, herbivory, and the resilience of coral reefs to climate change. Current Biology 17:360-365.
Module 5: What should we do?	M5P1: Langlois, J. et al. 2022. The aesthetic value of reef fishes is globally mismatched to their conservation priorities.<br>M5P2: Friedlander, A.M. et al. 2007. Coupling ecology and GIS to evaluate efficacy of marine protected areas in Hawaii. Ecology 17:715-730.<br>M5P3: Packer, C. et al. 2013. Conserving carnivores: Dollars and fence. Ecology Letters 16:635-641.<br>M5P4: Creel, S. et al. 2013. Conserving large populations of lions: The argument for fences has holes. Ecology Letters 16:1413-e3.

Introduction

Anthropogenic emissions of carbon dioxide (CO_2) and its uptake by the surface ocean cause profound changes in marine carbonate chemistry, including seawater acidification and lowering of the calcium carbonate saturation state [1], [2]. Contemporary surface ocean pH has decreased on average by 0.1 units due to CO_2 invasion since preindustrial times. According to IPCC projections atmospheric partial pressure of CO_2 (pCO_2) is expected to further increase from current ~390 µatm to ~760 µatm, corresponding to a drop in mean oceanic surface pH by 0.3 to 0.4 units until the end of the 21st century ('business-as-usual scenario' [3], [4]). This change in carbonate chemistry, termed ocean acidification (OA), is thought to primarily affect calcifying organisms building their shells and skeletons of calcium carbonate [5], [6], [7]. Biological effects of OA on non-calcifying organisms are diverse and often highly species-specific [8].

Our present understanding of potential OA impacts is almost entirely limited to single species responses, while OA consequences for food web interactions remain poorly understood. Indirect impacts through trophic interactions are expected because OA may change the biochemical composition of primary producers that affects nutritional food quality for consumers. Increased CO_2 can stimulate carbon fixation by photosynthetic organisms and thereby reduce the nutrient content relative to carbon [9], [10], [11], which determines the food quality for herbivores [12]. Enhanced carbon consumption relative to nutrients under elevated CO_2 conditions [13], [14] can cause an imbalance between phytoplankton stoichiometric composition and consumer nutrient demand for somatic growth [11]. Besides elemental stoichiometry, fatty acid (FA) associated food quality is a critical factor that regulates the energy transfer between primary producers and consumers [15], [16], because essential FAs cannot be synthesized *de novo* by heterotrophic organisms and have to be acquired through the diet. In particular long-chain polyunsaturated FAs (PUFAs) such as docosahexaenoic acid (DHA), eicosapentaenoic acid (EPA) and arachidonic acid (ARA) play an important role in growth, development and reproduction success in heterotrophs [17], [15]. OA may impact phytoplankton FA synthesis because extracellular pH is known to affect various intracellular physiological parameters [18] that influences enzyme activity.

The classic diatom-copepod-fish link in the ocean supports some of the most productive ecosystems in the world and is an important source of highly nutritious food for upper trophic levels.

Experimental studies indicate a weak sensitivity of primary production to CO_2 [14] and no direct effects on copepod growth and hatching success at CO_2 levels within the range expected by the end of this century [19], [20]. However, CO_2 may indirectly affect zooplankton growth through its potential impact on the nutritional quality of phytoplankton, their major food source. To test this hypothesis, we independently manipulated CO_2 concentration in both diatoms used as food algae and copepod cultures, and investigated dietary OA effects on copepod growth and reproduction. The experiment consisted of a two-by-two factorial design crossing two CO_2 levels in food algae media and seawater used for copepod growth. The cryptophyte *Rhodomonas* sp. and diatom *Thalassiosira pseudonana* were used as food source and the copepod *Acartia tonsa* as consumer. We determined resulting FA composition of both alga and copepod as well as copepod development and reproduction. Our experiment showed that elevated CO_2 affected biochemical composition of the diatom that constrained copepod growth performance.

Module: Global Climate Change

Paper: 3

Section: Methods and Results

[redacted]

Methods

CO_2 manipulation and experimental design

The target values for experimental CO_2 manipulation were 380 µatm for the low (L) and 740 µatm for the high (H) CO_2 treatment. Phytoplankton (P) was grown at both L and H pCO_2 concentrations and fed to copepod zooplankton (Z) grown in seawater at the same L and H target levels in a crossed design. It is important to note that biological activity, such as photosynthesis and respiration, alter the carbonate system. In addition, water exchange and combining treatments with low and high CO_2, as was done in the copepod growth experiment, can result in deviations from the target CO_2 levels. Nevertheless, pCO_2 levels of L and H treatments were maintained close to target values and differences among treatments persisted throughout the experiment (Figure S1).

Rhodomonas sp. and *T. pseudonana* were cultured as food sources in artificial seawater at pCO_2 of ~495±100 SD (L) and ~760±110 (H) for *Rhodomonas* sp. and ~365±120 (L) and ~915±270 (H) µatm for *T. pseudonana*, respectively. Juvenile copepods were fed with *Rhodomonas* to ensure optimal growth of the first developmental stages and *T. pseudonana* was used as food source after copepodite stage 1. The carbonate system of *T. pseudonana* cultures was manipulated by combined additions of sodium carbonate (Na_2CO_3) and hydrogen chloride (HCl) at constant alkalinity; the two CO_2 treatments for *Rhodomonas* cultures were continuously aerated with CO_2-enriched air. Algae were grown in laboratory batch cultures on a 18:6 light:dark cycle with replete nutrients. To investigate the response time of algae fatty acid composition alterations to changing pCO_2, *T. pseudonana* was grown at high (~1120 µatm) pCO_2 for five days and then transferred to a low (~380 µatm) pCO_2 media. FA concentration was measured every five hours over a 30 h time period.

Acartia tonsa eggs were hatched in seawater under pCO_2 conditions of ~380 µatm. After the nauplii reached developmental stage 2, they were transferred into 2-L NALGENE bottles (1000 individuals L^{-1}) filled with seawater (salinity 18.2) from a tank that was aerated continuously with appropriately CO_2-enriched air of ~495±100 (L) and ~760±110 (H) µatm pCO_2, respectively. Copepod zooplankton (Z) were fed with CO_2 preconditioned phytoplankton (P) at about 1000 µg C L^{-1} in a factorial design with four treatment combinations: P_L/Z_L, P_L/Z_H, P_H/Z_L and P_H/Z_H, each with three replicates. Water and food during the copepod growth experiment were replaced every other day. All replicates were randomly placed in a temperature-controlled culture room at 18°C and 14:10h light:dark cycle until the copepods reached adult stage. Over the course of the experiment, developmental stages were identified and at the end of the growth experiment egg production of females measured over 24 h and hatching success of eggs and nauplii morphological formation observed for two days. Species involved for this experiment were lab cultures and thus no specific permits were required for the sample collection.

Dissolved inorganic carbon (DIC) was measured after every water exchange and pH was recorded daily during the copepod growth experiment. [redacted] During the copepod growth experiment measured mean (±SD) pH values were 8.14±0.12 and 7.94±0.08, and for DIC 480±110 and 725±140 µatm CO_2 in the L and H treatment, respectively (Figure S1). DIC values in the crossed treatments were 485±80 (P_H/Z_L) and 745±80 (P_L/Z_H) µatm CO_2. Due to the fact that NBS based pH measurements are rather weak for reliable carbonate chemistry calculations, total alkalinity (TA) measurements (Text S1) were taken two times per week for crosscheck calculations. Values for pCO_2 calculated from pH and DIC differed from pCO_2 calculations using DIC and TA on average ~110 and ~210 µatm at the low and high CO_2 treatment level, respectively, over the duration of the experiment. These uncertainties are probably higher than the real error since they were caused by outliers in TA and DIC measurements to which the carbonate system is relative insensitive when pH is involved in the calculations.

FA composition of *T. pseudonana* was analyzed from the stock culture during exponential growth phase and of copepod females at the end of the experiment. FAs were measured as fatty acid methyl esters (FAMEs) with a Thermo GC Ultra gas chromatograph [redacted]

Statistical analysis

Algal responses to experimental conditions were assessed using two-tailed *t*-tests. Differences in copepod FA classes and egg production between treatments were tested using analysis of variance (ANOVA). A Tukey HSD post hoc test was used to assess differences among treatments in egg production. Generalized linear models (GLM) were used to examine the effect of the seawater pCO_2 used for algal and copepod cultures on the relative proportion of FA classes in copepods. Principal component analysis (PCA) was used to assess the difference in individual FA composition of the diet algae and copepods across the treatment combinations. For algal food, log-transformed FA concentration per cell and for copepods arcsine-square root transformed percentage of total FA was used since the proportion of FA classes varies between *T. pseudonana* and *A. tonsa* (see Figure 1). Each FA was standardized by subtracting its mean and dividing by its standard deviation, assembling the resulting standardized series into a 15-FA by 20-treatment combination data matrix. [redacted] This analysis identified FA that explained most to the observed variance. Statistical analyses were performed using Statistica and the R software environment 2.14.1 [23].

Note: Results and Discussion are combined in this paper, so only the figures and table are shown here.

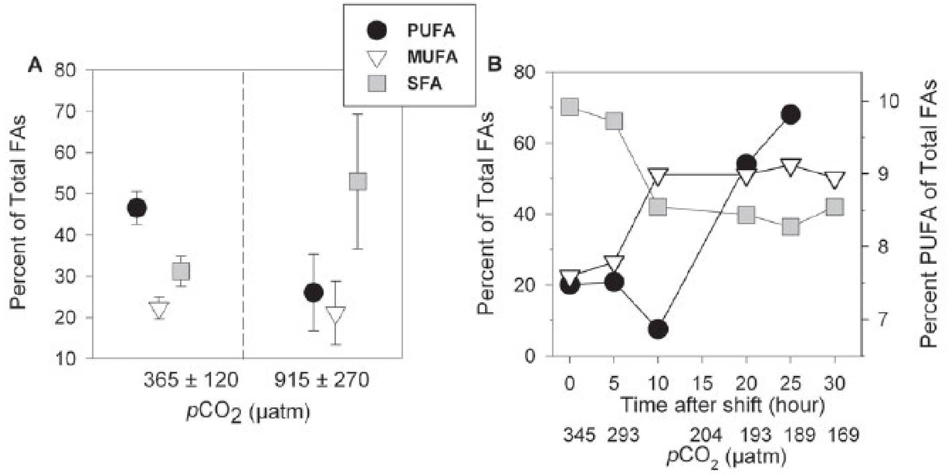

Figure 1. Fatty acid composition and concentration of *Thalassiorira pseudonana* cultured at different CO_2 treatments. A) Percentage of polyunsaturated (PUFA), monounsaturated (MUFA), and saturated (SFA) fatty acids relative to total fatty acids during the exponential growth phase cultured at low (realized value of 365 µatm pCO_2, n = 5) and high (realized value of 915 µatm pCO_2, n = 3) CO_2 treatments used as copepod food source. **B**) Change in the fatty acid composition in *T. pseudonana* after a shift from high to low pCO_2 conditions (n = 1 per treatment level). Time 0 are measured values before the culture media shift. Error bars indicate standard errors.
doi:10.1371/journal.pone.0034737.g001

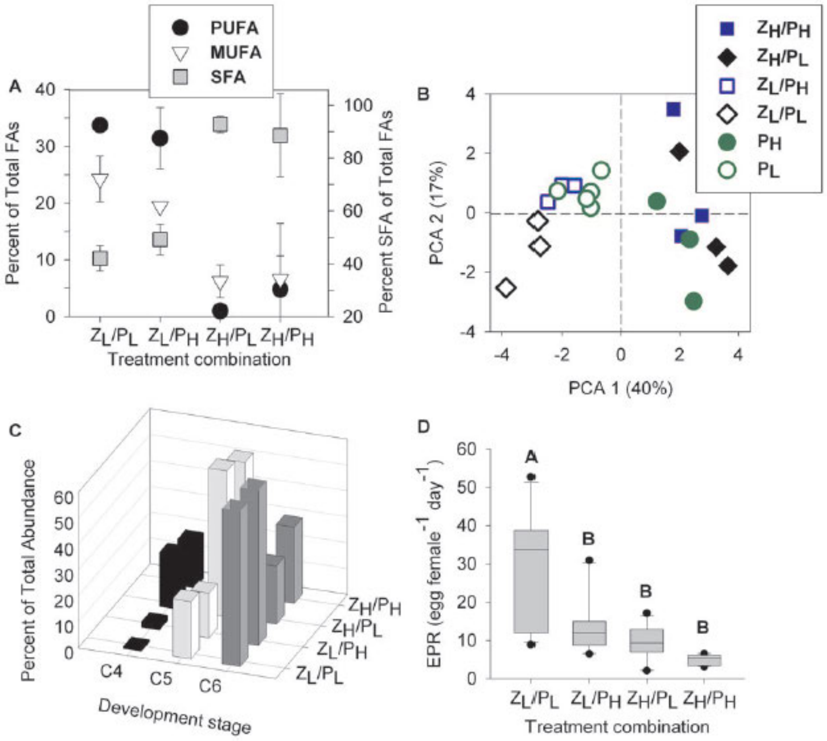

Figure 2. Fatty acid composition, somatic growth and reproduction of *Acartia tonsa* across CO_2 treatment combinations. A) Percentage of polyunsaturated (PUFA), monounsaturated (MUFA), and saturated (SFA) fatty acids relative to total fatty acids in female copepods. **B**) Principal component analysis (PCA) of fatty acid composition for the dietary algae *Thalassiorira pseudonana* and *A. tonsa* of the different treatment combinations. PCA scores 1 explained 40% of the variability (see *x*-axis of c) and was highly negatively correlated with 22:6*n*-3 ($r^2 = 0.73$), 20:4*n*-6+20:5*n*-3 ($r^2 = 0.85$), 18:3*n*-6 ($r^2 = 0.73$) and 16:1 ($r^2 = 0.79$), and positively with 22:1*n*-9 ($r^2 = 0.25$) and 18:1*n*-9*t* ($r^2 = 0.57$). PCA score 2 explained 17% of the overall variability (see *y*-axis of c) and was strongest positively correlated with 24:0 ($r^2 = 0.84$). Loadings of the PC scores are shown in Figure S2). **C**) Stage distribution of *A. tonsa* individuals at day 10. C4, C5, C6 = copepodite stage 4, 5, and adult, respectively. **D**) Egg production rate (EPR) of incubated females (n = 12 per treatment level). EPR was significantly different between treatments ($F_{(3, 44)} = 18.02$, $p < 0.001$). Different letters above bars represent significant differences from a Tukey HSD test. The bars represent the 25th, 50th and 75th percentiles, whiskers stand for the 10th and the 90th percentiles and black points show outliers. Legend refers to treatment combinations of copepod zooplankton (Z) and phytoplankton food source (P) at low (L) and high (H) pCO_2.
doi:10.1371/journal.pone.0034737.g002

Table 1. Regression statistics of *Acartia tonsa* egg production as a linear function of fatty acid composition.

Fatty acid	Slope	*Y*-intercept	r^2	*p-value*
PUFA (%)	0.03	1.9	0.52	0.013
MUFA (%)	0.07	1.6	0.73	**<0.001**
SFA (%)	−0.02	3.8	0.60	**0.005**
PUFA:SFA	1.3	1.9	0.59	**0.006**
ARA-EPA (ng cop^{-1})	0.68	2.06	0.67	**0.002**
DHA (ng cop^{-1})	1.23	1.92	0.77	**<0.001**

Bonferroni-corrected significance levels for multiple fatty acid comparisons were $\alpha = 0.008$ (0.05/6). Significant correlations are highlighted in bold; n = 11. PUFA = polyunsaturated fatty acid; MUFA = monounsaturated fatty acid; SFA = saturated fatty acid; ARA-EPA = 20:5*n*3; DHA = docosahexaenoic acid (22:6*n*3).

doi:10.1371/journal.pone.0034737.t001

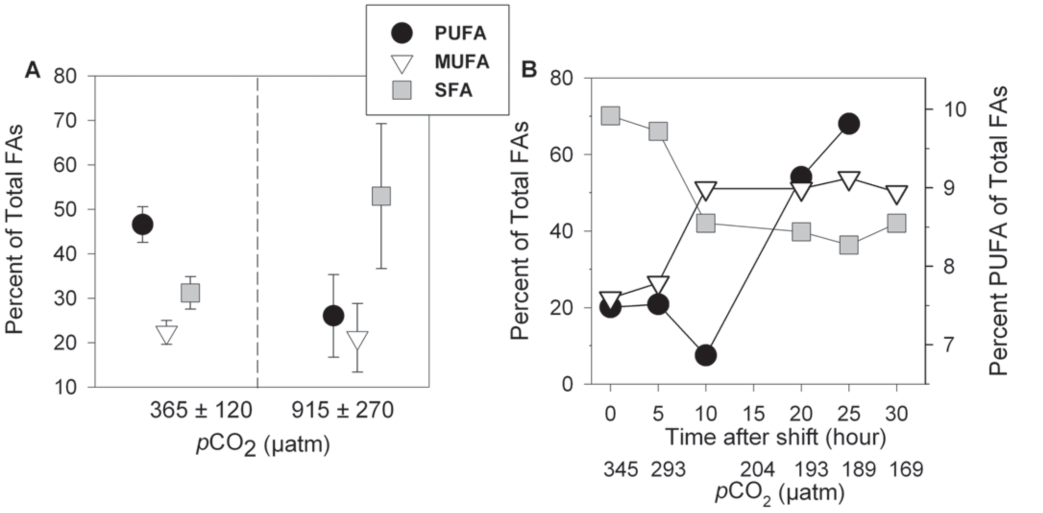

Figure 1. Fatty acid composition and concentration of *Thalassiorira pseudonana* cultured at different CO_2 treatments. A) Percentage of polyunsaturated (PUFA), monounsaturated (MUFA), and saturated (SFA) fatty acids relative to total fatty acids during the exponential growth phase cultured at low (realized value of 365 µatm pCO_2, n = 5) and high (realized value of 915 µatm pCO_2, n = 3) CO_2 treatments used as copepod food source. **B**) Change in the fatty acid composition in *T. pseudonana* after a shift from high to low pCO_2 conditions (n = 1 per treatment level). Time 0 are measured values before the culture media shift. Error bars indicate standard errors.
doi:10.1371/journal.pone.0034737.g001

Results and Discussion

Our experiment showed that CO_2 concentration significantly changed FA concentration and composition in the diatom *T. pseudonana* used for copepod diet. The relative amount of PUFAs was significantly lower (t 4.48, p 0.004) and the amount of SFAs higher (t 3.37, p 0.015) at high pCO_2 compared to the low pCO_2 treatment (Figure 1A). Essential PUFA concentrations were significantly reduced at high pCO_2 (Table S1), specifically DHA (22:6n 3; t 2.81, p 0.03) and the group ARA EPA (20:4n 6, 20:5n 3; t 6.63, p<0.001). A shift in FA composition at projected future CO_2 levels is consistent with observations in the coccolithophorid *Emiliania huxleyi* [24] and with green algae and prymnesiophyte experiments conducted at extreme CO_2 changes [25], [26], [27].

A separate experiment confirmed that the shift in FA occurred rapidly in response to changing pCO_2 in the diatom *T. pseudonana.* When transferred from high to low CO_2, FA composition was already significantly different from its initial composition after 15 h (Figure 1B) and FA components changed in the same direction as observed at constant high and low pCO_2 treatments. Similarly, a rapid transition in FA composition can be expected when algae are transferred from low to high pCO_2, which was, however, not tested in our experiment. Though, a rapid reversible FA response to changing pCO_2 concentration has been reported in green algae [26]. The higher unsaturation levels of FAs in algae cells cultured at low pCO_2 compared to cells at high pCO_2 has been suggested to be partially a consequence of repressed FA synthesis, which promotes the desaturation of pre existing SFAs [26]. Recently it has been proposed that pH might act as a regulation signal for the formation of cell membranes, which are mainly composed of fatty acids, by controlling the production of its synthesizing enzymes [28]. A high environmental pCO_2 (low pH) can decrease the internal cell pH [29]. Therefore the increased amount of SFAs could be a mechanism to control the internal cell pH, as a membrane built of short chain FAs is less fluid and permeable to CO_2. However, the cellular processes involved in FA synthesis under changing pH or pCO_2 levels are not fully understood.

Similar to FA modification in algal food, FA concentration and composition of adult copepods varied significantly between CO_2 treatments. The mean ±SD total amount of FAs in *A. tonsa* was significantly different across treatments ($F_{(3,\ 8)}$ 5.15, p 0.028) and higher when raised and fed with algae cultured at low pCO_2, with 8.9±5.6 ng ind. ^{1} compared to 0.8±0.2 ng ind. ^{1} when both copepods and algal diet were cultured at high pCO_2 and to 2.3±0.5 ng ind. ^{1} in the crossed treatment combinations (Table S1). Copepods raised and fed with algae at low pCO_2 contained high proportions of PUFAs relative to total FAs that are in the same range with reports in marine calanoids [30]. The PUFA fraction in copepods decreased from more than 30% at low pCO_2 to less than 5% at high pCO_2 ($F_{(3,\ 8)}$ 54.51, p<0.001) (Figure 2A). The long chain highly unsaturated FAs DHA and ARA EPA, which are important components for growth and reproduction of consumers [31], decreased from 15% in copepods raised at low pCO_2 below detection limit in those at high pCO_2 (Table S1). Similarly, the proportion of MUFAs (monounsaturated fatty acids) varied significantly across treatments ($F_{(3,\ 8)}$ 8.2, p 0.008) and decreased from around 20% at low pCO_2 to less than 10% at high pCO_2. On the other hand, the relative amount of SFAs tripled in copepods at high pCO_2 (Figure 2A) and FA compositions were different between treatments ($F_{(3,\ 8)}$ 26.22, p <0.001).

Contrary to our expectation, FA composition in copepods differed between individuals raised at low and high seawater pCO_2, irrespective of the CO_2 level of their algal diet (Figure 2A). Because consumers are unable to synthesize PUFAs we expected that copepod FA composition in the crossed pCO_2 treatments of copepod culture and food algae (P_L/Z_H, P_H/Z_L) would reflect changes in FA of their diet. Principal component analysis (PCA) of individual FAs in diet algae and copepods also showed distinctive clustering of the copepod groups raised at low and high pCO_2 treatments, irrespective of the CO_2 conditions of their diet algal culture (Figure 2B), which was mainly explained by PUFAs and SFAs (Figure S2). A GLM model supported that CO_2 concentra tion of the seawater used to raise copepods significantly negatively affected the relative proportion of PUFAs (p<0.001) and positively affected the proportion of SFAs in copepods (p<0.001), which was

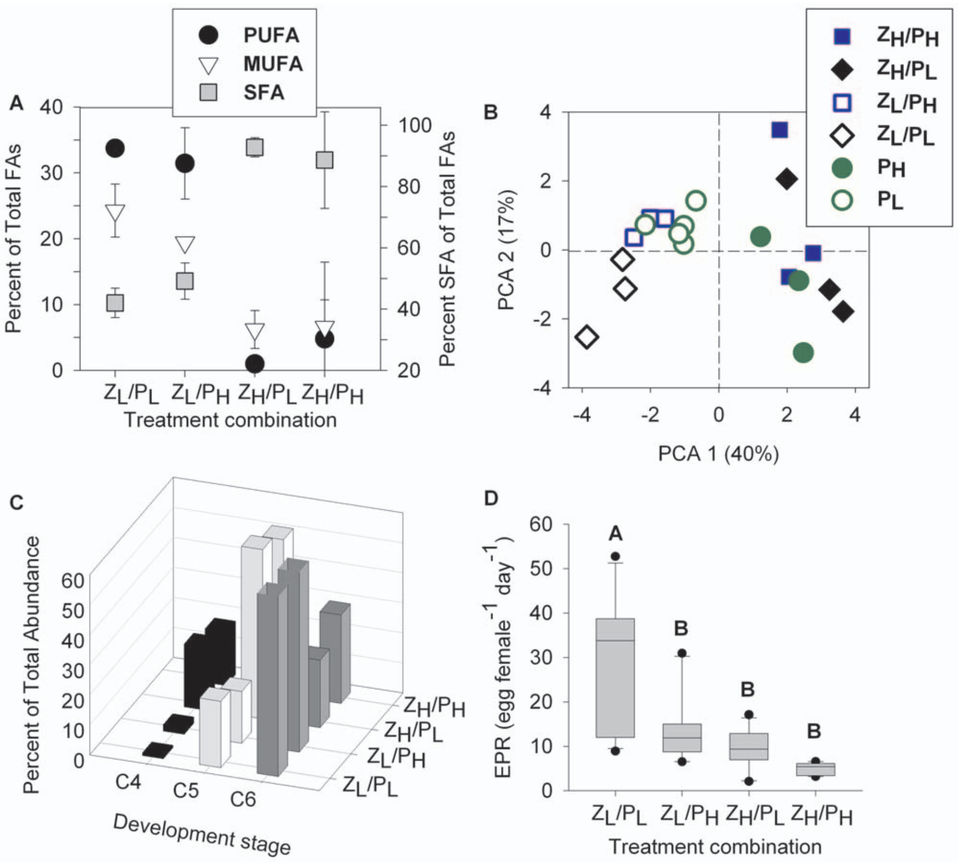

Figure 2. Fatty acid composition, somatic growth and reproduction of *Acartia tonsa* across CO_2 treatment combinations. A) Percentage of polyunsaturated (PUFA), monounsaturated (MUFA), and saturated (SFA) fatty acids relative to total fatty acids in female copepods. **B**) Principal component analysis (PCA) of fatty acid composition for the dietary algae *Thalassiorira pseudonana* and *A. tonsa* of the different treatment combinations. PCA scores 1 explained 40% of the variability (see *x* axis of c) and was highly negatively correlated with 22:6*n* 3 ($r^2 = 0.73$), 20:4*n* 6+20:5*n* 3 ($r^2 = 0.85$), 18:3*n* 6 ($r^2 = 0.73$) and 16:1 ($r^2 = 0.79$), and positively with 22:1*n* 9 ($r^2 = 0.25$) and 18:1*n* 9*t* ($r^2 = 0.57$). PCA score 2 explained 17% of the overall variability (see *y* axis of c) and was strongest positively correlated with 24:0 ($r^2 = 0.84$). Loadings of the PC scores are shown in Figure S2). **C**) Stage distribution of *A. tonsa* individuals at day 10. C4, C5, C6 = copepodite stage 4, 5, and adult, respectively. **D**) Egg production rate (EPR) of incubated females (n = 12 per treatment level). EPR was significantly different between treatments ($F_{(3, 44)} = 18.02$, $p < 0.001$). Different letters above bars represent significant differences from a Tukey HSD test. The bars represent the 25th, 50th and 75th percentiles, whiskers stand for the 10th and the 90th percentiles and black points show outliers. Legend refers to treatment combinations of copepod zooplankton (Z) and phytoplankton food source (P) at low (L) and high (H) pCO_2.
doi:10.1371/journal.pone.0034737.g002

consistent across combinations and not dependent on the pCO_2 level of the algal culture.

These findings suggests that the FA composition of algae changed rapidly when transferred from low pCO_2 culture media to high pCO_2 seawater used to raise copepods and *vice versa*. Since consumers are unable to synthesize PUFAs [30] and previous experiments showed that copepod growth is rather insensitive to CO_2 levels within OA predictions [19], [20], direct CO_2 effects on copepod FA synthesis seem unlikely. In our experiment, water and food was exchanged every second day and algae were in their exponential growth. Thus, we rather expect that high turnover rates and the ability of *T. pseudonana* to rapidly change the FA composition in a variable pCO_2 environment (Figure 1B) are responsible for an adjustment in FA composition in the crossed treatments within the first day. Rapid modification in algae FA and the fact that *A. tonsa* has no lipid reserves [32] likely explains the absence of the influence from the algae culture media pCO_2 on copepod FA composition within both crossed treatment combi nations.

The CO_2 dependent dietary shift in FAs had a significant effect on *A. tonsa* growth and development. Copepods of the same age (10 d) showed a delay in stage development of 1 to 2 days at high pCO_2 (Figure 2C). Egg production decreased from a median of 34 eggs female ^{1} d ^{1} at low water and food pCO_2 to less than 12 eggs female ^{1} d ^{1} in all other treatments, with the lowest production (5 eggs female ^{1} d ^{1}) at high water and food pCO_2 (Figure 2D). The egg production rate was significantly related to the ratio of PUFA:SFA and the content of DHA and ARA EPA within the female copepods (Table 1), consistent with other observations in zooplankton [33]. Copepod egg production raised at low pCO_2 and fed with algae grown at high pCO_2 produced significantly less eggs compared to copepods in the low pCO_2 treatment combination (Figure 2D). This significant decline is most likely a result of the overall lower copepod FA quantity when fed with algae cultured at high CO_2 compared to food at low CO_2 (Table S1). Given that adult *A. tonsa* females invest the majority of their lipids into reproduction [34], the significant decrease of essential PUFAs due to low quality food algae is most likely the reason for

Table 1. Regression statistics of *Acartia tonsa* egg production as a linear function of fatty acid composition.

Fatty acid	Slope	*Y*-intercept	r^2	*p value*
PUFA (%)	0.03	1.9	0.52	0.013
MUFA (%)	0.07	1.6	0.73	**<0.001**
SFA (%)	0.02	3.8	0.60	**0.005**
PUFA:SFA	1.3	1.9	0.59	**0.006**
ARA EPA (ng cop^{-1})	0.68	2.06	0.67	**0.002**
DHA (ng cop^{-1})	1.23	1.92	0.77	**<0.001**

Bonferroni corrected significance levels for multiple fatty acid comparisons were $\alpha = 0.008$ (0.05/6). Significant correlations are highlighted in bold; n = 11. PUFA = polyunsaturated fatty acid; MUFA = monounsaturated fatty acid; SFA = saturated fatty acid; ARA EPA = 20:5*n*3; DHA = docosahexaenoic acid (22:6*n*3).

doi:10.1371/journal.pone.0034737.t001

the considerable decline in egg production observed in the high pCO_2 treatment combinations (Figure 2).

[redacted]

Our study suggests that OA can have important consequences for consumer growth and production by affecting the nutritional quality of primary producers that translates to higher trophic levels. These results are consistent with experiments on freshwater cladocerans, fed with algae from an acidic lake [37], suggesting that our results are not restricted to monospecific laboratory cultures and may be expected at community level. However, future experimental manipulations are required to clarify the widespread response of phytoplankton biochemical composition to ocean acidification at relevant pCO_2 levels in other taxonomic groups and natural communities. It can be expected that trophic upgrading and differential algae sensitivity to pCO_2 at the community and ecosystem level may compensate for low food quality observed at the single species level. Moreover, the tolerance to pCO_2 and pH might be lower for monocultures compared to natural populations, which have high ecophysiolog ical variability [38] and genetic diversity, important for adaption to various environmental factors [39]. Nonetheless, shifts in FA composition as a response to changing CO_2 have been documented in other phytoplankton species [26], [40], and FA responses in phytoplankton as observed here might be important during bloom periods if CO_2 sensitive organisms dominate.

The effect of OA on nutritional quality in the diatom copepod food chain relationship observed in our study may have far reaching consequences for food webs since FAs originating in phytoplankton are sequentially incorporated into the total lipid fraction of zooplankton and triacylglycerol of larval fish [41]. Given that fish is a critical natural resource [42], acidification driven food quality deterioration may impair fish production by changing the biochemical composition of food algae and its transfer to higher trophic levels [43], [44]. While it is difficult to extrapolate from monocultures to community level, these results point to the likelihood that OA consequences go beyond direct physiological impacts and that indirect effects through trophic interactions need to be considered.

[redacted]

[redacted]

[redacted]

Table S1. Results of t-tests for the pre- and post-course Eco/Evo MAPS assessment based on whether students had taken AP Bio (A) and one-way ANOVAs based on their class year (B). In header rows, numbers in parentheses equal sample sizes, and in the table body, numbers in parentheses equal 1 S.E. 2016 and 2018 students are combined.

A	No AP Bio (11)	AP Bio (26)	t	df	P
Pre-course average	71.4 (3.1)	74.8 (2.3)	0.91	22	0.37
Post-course average	78.4 (3.0)	79.0 (2.2)	0.17	20	0.86
Post – Pre difference	6.8 (2.0)	5.5 (1.1)	1.2	15	0.25

B	Sophomore (9)	Junior (14)	Senior (14)	$F_{2,34}$	P
Pre-course average	69.7 (3.7)	77.1 (3.0)	73.2 (3.0)	1.26	0.30
Post-course average	78.1 (3.6)	81.1 (2.9)	77.0 (2.9)	0.53	0.59
Post – Pre difference	8.5 (1.9)	4.0 (1.5)	3.7 (1.5)	2.20	0.13
